# Single-cell RNA sequencing reveals myeloid and T cell co-stimulation mediated by IL-7 anti-cancer immunotherapy

**DOI:** 10.1038/s41416-024-02617-7

**Published:** 2024-02-29

**Authors:** Hye Hyeon Eum, Dasom Jeong, Nayoung Kim, Areum Jo, Minsu Na, Huiram Kang, Yourae Hong, Jin-Sun Kong, Gi Heon Jeong, Seung-Ah Yoo, Hae-Ock Lee

**Affiliations:** 1 Department of Microbiology, College of Medicine, The Catholic University of Korea, Seoul, 06591 Republic of Korea; 2 Department of Biomedicine and Health Sciences, Graduate School, The Catholic University of Korea, Seoul, 06591 Republic of Korea; 3 Digestive Oncology, Department of Oncology, Katholieke Universiteit Leuven, Leuven, Belgium; 4 Center for Integrative Rheumatoid Transcriptomics and Dynamics, College of Medicine, The Catholic University of Korea, Seoul, 06591 Republic of Korea

**Keywords:** Cancer immunotherapy, Cancer microenvironment, Recombinant protein therapy, Next-generation sequencing, Cytological techniques

## Abstract

**Background:**

Immune checkpoint inhibitors unleash inhibitory signals on T cells conferred by tumors and surrounding stromal cells. Despite the clinical efficacy of checkpoint inhibitors, the lack of target expression and persistence of immunosuppressive cells limit the pervasive effectiveness of the therapy. These limitations may be overcome by alternative approaches that co-stimulate T cells and the immune microenvironment.

**Methods:**

We analyzed single-cell RNA sequencing data from multiple human cancers and a mouse tumor transplant model to discover the pleiotropic expression of the Interleukin 7 (IL-7) receptor on T cells, macrophages, and dendritic cells.

**Results:**

Our experiment on the mouse model demonstrated that recombinant IL-7 therapy induces tumor regression, expansion of effector CD8 T cells, and pro-inflammatory activation of macrophages. Moreover, spatial transcriptomic data support immunostimulatory interactions between macrophages and T cells.

**Conclusion:**

These results indicate that IL-7 therapy induces anti-tumor immunity by activating T cells and pro-inflammatory myeloid cells, which may have diverse therapeutic applicability.

## Introduction

Immune checkpoint inhibitors (ICIs) targeting Programmed Cell Death Protein 1 (PD-1) or Programmed death-ligand 1 (PD-L1) are immunotherapeutic drugs used to treat solid cancers. Despite the remarkable therapeutic success of PD-1/PD-L1 inhibitors, many cancer patients do not respond to ICI treatment and require alternative options. PD-L1 expression on tumor cells facilitates FDA-approved companion diagnostics for PD-1/PD-L1 inhibitors [1], and the abundance of PD-1^+^ T cells elicits a better clinical response [2, 3], indicating that the expression of target molecules is a critical determinant of the therapeutic response. ICI responsiveness is not solely determined by checkpoint expression but also by antigenicity and other tumor features, as well as immunomodulatory cells and molecules [4]. Therefore, alternative or combination strategies have been proposed to enhance tumor immunogenicity or overcome various immunosuppressive mechanisms [5].

Active immunization is required to elicit anti-tumor immunity in tumors with insufficient infiltrating immune cells, whereas immunomodulatory strategies are appropriate for immune cell-enriched tumors. High-throughput analyses of human cancer using single-cell RNA sequencing (scRNA-seq) or mass cytometry have revealed various immune targets at an absolute expression level, as well as from an inferred interaction network of immune cells, thereby facilitating an unprecedented discovery of novel immunotherapeutic strategies [6–8]. Previous analyses reveal that regulatory T cells (Tregs) and exhausted CD8^+^ T cells are abundant in tumor tissues [6, 9–11]. When combined with PD-1 inhibitors, targeting inhibitory molecules on these cell types, such as CTLA-4 or LAG-3, had proven effective [12, 13]. Myeloid cells, including macrophages and dendritic cells (DCs), are an additional prominent component of the tumor microenvironment (TME) that can either promote or inhibit tumor growth [7, 14]. TREM2-expressing macrophages are known to foster a suppressive immune microenvironment, and TREM2 inhibition attenuates tumor growth in a preclinical model when combined with PD-1 inhibitors [15].

The Interleukin 7 (IL-7) pathway is known to support the development and maintenance of lymphoid populations such as T cells, B cells, and NK cells [16]. Based on preclinical studies which corroborated that pharmacological administration of IL-7 induces T cell expansion with minimal toxicity, cancer has been investigated as potential therapeutic targets of IL-7 agonists [16, 17]. The first report on the administration of recombinant human IL-7 (rhIL-7) to tumor patients was published in melanoma in 2006 [18]. Currently, NeoImmuneTech is orchestrating phase I/II studies of rhIL-7-hyFc (Efineptakin alfa, NT-I7) in multiple tumors including solid tumors, glioma, and large B cell lymphomas. Most clinical trials of IL-7 in tumors have been designed for synergistic administration with checkpoint inhibitors or chemoradiation, indicating the efficacy of IL-7 as an adjuvant therapy.

In this paper, we analyzed scRNA-seq data from human cancers and a mouse tumor transplant model and assessed the treatment efficacy of recombinant IL-7 (rIL7) in a mouse model. This is the first study examining multicellular effects of rIL7 anti-cancer therapy at single-cell resolution. The multicellular effects of rIL7 treatment highlight its therapeutic potential, which will broaden the options for immunotherapeutic strategies for cancer patients.

## Methods

### Analysis of human multi-cancer scRNA-seq data

The UMI gene-cell-barcode matrix derived from scRNA-seq data for lung adenocarcinoma (11 tumor and 11 normal) [19], colorectal cancer (23 tumor and 10 normal) [8], renal cell carcinoma (16 tumor and 15 normal) [20], and pancreatic ductal adenocarcinoma (24 tumor and 10 normal) [21] was downloaded from the NCBI Gene Expression Omnibus (GEO) and Genome Sequence Archive databases. Two quality measures were necessitated for each cell: mitochondrial genes (<20%) and gene counts (between 200 and 8000). The UMI counts for the genes in each cell were log-normalized using the *NormalizeData* function of the Seurat v3.1.5 R package [22] in R v3.6.3. Among cells that met the quality criteria, CD45^+^ cells with gene expression of *PTPRC* > 1 and *EPCAM* = 0 were selected for subsequent analysis. A total of 2000 variably expressed genes were selected using the *FindVariableFeatures* function with default option and the relative expression levels were defined with the *ScaleData* function to perform principal component analysis (PCA) by *RunPCA*. We used the *RunHarmony* function of the Harmony v1.0 R package [23] for batch correction. Cell clustering and UMAP visualization were performed using the *FindClusters* and *RunUMAP* functions of Seurat with specific parameters (31 PCs and 0.6 resolution for total cells). The identity of each cell cluster was annotated using the expressions of known marker genes and cluster-specific genes. Differentially expressed genes for each cluster were calculated using the *FindAllMarkers* function of Seurat (only.pos = TRUE, min.pct = 0.25, and test.use = “wilcox”).

### Cell line culture

The murine colon cancer cell line CT-26 (KCLB, 80009) was purchased from the Korean Cell Line Bank (Seoul, Korea) and cultured in Dulbecco’s Modified Eagle Medium (DMEM) (Gibco, 11965092) with 10% fetal bovine serum (Gibco, 16000044) supplementation. All the cultures were maintained in a humidified incubator with 5% CO_2_.

### Syngeneic mouse tumor model

Female BALB/c mice (5–6-week old) were purchased from Orient Bio (Seongnam, Korea) and used after a week of quarantine and stabilization. Tumors were developed by subcutaneous inoculation with 2 × ${10}^{5}$ CT-26 cells in 0.1 ml of PBS. Tumor volume was determined by measuring the vertical diameter of each tumor and calculating as follows: (A × B²)/2 (A = length of major axis, B = length of minor axis), and the mean of both sides. The mean ± SEM tumor volumes were expressed for all tumors formed in each experimental group. To investigate the anti-cancer effect of IL-7, they were divided into two groups (control and rIL7) with comparable mean tumor sizes. Mice with no visible tumor or with tumors larger than 200 mm^3^ in size at post-transplant day 8 were excluded from further analysis. Recombinant mouse IL-7 protein (RnD Systems, 407-ML/CF; 10 μg/0.1 ml) was intraperitoneally injected into tumor-bearing mice on days 11 and 13 post-inoculation of tumor cells, and the control group was treated with PBS. Tumors were collected within 2–3 weeks, depending on the health of the mice. All experimental procedures conformed to the IACUC guidelines for the care and use of laboratory animals established by the Catholic University of Korea (approval number: 2020-0239-01).

### Preparation of single cell isolates from mouse tissues

Tissues isolated from the mice were dissociated using a Tumor Dissociation Kit, mouse (Miltenyi Biotec, 130-096-730). According to the manufacturer’s recommendations, small pieces of tissue were incubated with an enzyme mix at 37 °C in a thermal mixer and agitated at 800 rpm for 40 min. Digested tissue was filtered using a 70-µm cell strainer, and red blood cells were lysed with an RBC lysis solution (QIAGEN, 158904). Live cell concentration was determined using AO/PI dye (Logos Biosystems, F23001), and then the single-cell isolates were subjected to further experiments, either pooled or individually. Number of mice for specific experiments are indicated in the Figure Legends.

### scRNA-seq and scTCR-seq for mouse tumor model

Cells isolated from tissues were pooled from 4 mice, sieved through a 40-µm cell strainer and loaded into the Chromium system (10x Genomics, Pleasanton, CA, USA). Total RNA capture, cDNA amplification, and library construction were conducted according to the manufacturer’s instructions using Single Cell 3ʹ GEM, Library & Gel Bead Kit v3 (10x Genomics, 1000075) or Next GEM Single Cell 5ʹ Library & Gel Bead Kit v1.1 (10x Genomics, 10000165). Prior to library construction, 5ʹ TCR transcripts were enriched for TCR profiling using the Single Cell V(D)J Enrichment Kit, Mouse T Cell (10x Genomics, 1000071). The generated mRNA and TCR libraries were sequenced using Illumina HiSeq2500 and NovaSeq6000 systems. Barcode processing and gene counting were performed using the CellRanger software v3.1.0 with the mm10-3.0.0 reference genome or the v5.0.0 multi pipeline with mm10-2020-A and vdj-GRCm38-alts-ensembl-5.0.0 reference genomes. Filtered feature matrices for mRNA profiles and a filtered contig list for TCR repertoire generated by CellRanger were used for the analysis.

### Analysis of mouse scRNA-seq data

Prior to analysis, two quality criteria were applied to each cell: <20% mitochondrial genes and >200 detected genes. In the case of the rIL7-treated dataset (5ʹ scRNA-seq), DecontX (celda v1.5.11) [24] was additionally performed to remove ambient RNA-contaminated cells because cells having mixed cell type features were highly observed. The gene expression matrix for the remaining cells was processed using the Seurat v3.1.5 R package. The UMI count was log-normalized, 2000 variably expressed genes were selected, and the relative expression levels of variably expressed genes were determined. The cell-cycle scores (S.score and G2M.score) were regressed during data scaling using *ScaleData* function. Cell-cycle scoring was accomplished by converting human cell-cycle genes (“cc.genes”) from the Seurat R package into mouse gene symbols using the *useMart* and *getLDS* functions of the biomaRt R package, and then calculating the S.score and G2M.score with the *CellCycleScoring* function. PCA was followed by batch correction, cell clustering, and UMAP visualization. The cell type in each cluster was annotated based on the expression of known marker genes. To identify differentially expressed genes for each cluster or group, the *FindAllMarkers* function was used with the default options.

### Pseudotime estimation for T cells

The Monocle (version 3) algorithm was used to estimate the cell order (pseudotime) for the state transitions of T cells [25]. The UMI gene-cell-barcode matrix was provided as input to Monocle, and its *new_cell_data_set* function was used to create the object. The *preprocess_cds* function was used to normalize the expression value, and the *align_cds* function was used to correct the batch effects. The UMAP coordinates were obtained from the results obtained by Seurat. The cell trajectory was inferred using *cluster_cells*, *learn_graph*, and *order_cells* functions with default parameters.

### Signature scoring of tumor immune cells

Signature scores for CD4 and CD8 T cells were assessed by averaging the log-normalized expression levels of the following gene sets: naiveness (CCR7, SELL, LEF1, and TCF7 for humans; orthologs for mice), exhausted (CTLA4, HAVCR2, LAG3, PDCD1, and TIGIT for humans; orthologs for mice), and cytotoxicity (GZMA, GZMB, IFNG, NKG7, PRF1, GNLY, and GZMH for humans; Gzma, Gzmb, Ifng, and Nkg7 for mice). Alternatively, the cytotoxicity and dysfunctionality of CD8 T cells were measured using the *AddModuleScore* function of the Ucell v1.3.1 R package [26] based on the Mann–Whitney U statistic using the following gene sets: cytotoxicity (Gzmb, Fasl, Prf1, Ifng, Klrc1, Gzmk, and Xcl1) and dysfunction (Pdcd1, Ctla4, Havcr2, Entpd1, Vsir, Tox, and Eomes). To evaluate the M1/M2 scores of the macrophages, we used public gene sets obtained from in vitro and in vivo M1 and M2 cells [27]. The M1/M2 scores were calculated by averaging the log-normalized expression levels for 555 genes from in vitro M1, 340 genes from in vitro M2, 290 genes from in vivo M1, and 175 genes from in vivo M2. In the M1 vs M2 scatter plots, ellipses were calculated using the *stat_ellipse* function and linear regression analysis was performed using the *stat_smooth(method* = *”lm”)* function of the ggplot2 R packages.

### Analysis of TCR repertoire using scTCRseq data

TCR profiling was accomplished using scRepertoir v1.1.2 R package [28]. First, filtered contigs from the CellRanger output were combined using the *combineTCR* function. Only the alpha-beta chain pairs detected in the T cells were selected. Next, we used *combineExpression(cloneCall* = *”gene+nt”)* to determine clonotypes based on combinations of nucleotide and gene sequences and then calculated clonotype frequencies in each sample. Furthermore, *occupiedscRepertoire*, *clonalHomeostasis*, and *alluvialClonotypes* functions were used to visualize the clonotype frequencies. We defined the clonotype group as single =1, small =2, medium =3, large ≤5, and hyperexpanded >5.

### Prediction of cellular networks

Prediction of cellular networks, including inter- and intracellular interactions, was performed using CellphoneDB [29] using scRNA-seq data. We executed CellphoneDB with a threshold of 0.25 using normalized UMI counts in Python v3.8.5. Mouse genes were converted to human genes based on ortholog ensemble IDs using the biomaRt R package, and minor cell types comprising less than 1% of the total cells were excluded. Only ligands and receptors expressed in >25% of the cells in each subset were used for analysis. The results were visualized using the ggplot2, circlize, and migest R packages. The top five frequently interacting pairs are listed, but six pairs were selected in the rIL7 group because both fibroblast-NK cells and macrophage-NK cells ranked fifth.

To investigate the ligand-target links activated between macrophages and T cells after rIL7 treatment and their regulatory potential, we used the NicheNet algorithm [30]. The NicheNet R package was used with condition_oi = “rIL7”, condition_reference = “Control”, and organism = “mouse”. Ligand-target pairs with log2-foldchange in ligand expression >0.1, log2-foldchange in target expression >0.2, and regulatory potential >0 were selected as remarkable links.

### Spatial transcriptome profiling

Fresh tissues were embedded in optimal cutting temperature (OCT) compound (Sakura Finetek, 4583) and snap-frozen in 2‐methyl butane according to the 10x Genomics’ protocol for Visium Tissue Preparation. Spatial transcriptome data were obtained from GENINUS Inc. using the Visium Spatial Gene Expression Slide & Reagents Kit v1 (10x Genomics, 1000184). Briefly, the OCT-embedded tissues were cryosectioned to a thickness of 10-µm and Hematoxyling and Eosin (H&E) stained on a Visium Spatial Gene Expression Slide, followed by permeabilization, reverse transcription, second strand synthesis, denaturation, and cDNA amplification. Libraries were sequenced using the Illumina NovaSeq6000 system. Barcode processing and gene counting for each spot were performed using SpaceRanger v1.1.0 with the mm10-2020-A reference genome.

To evaluate the cell type composition of each spot, we used SPOTlight, a seeded non-negative matrix factorization (NMF) regression algorithm [31]. We excluded “Aggregates” or “Unknown” cells from the scRNA-seq data before use. Spots that did not meet the following conditions were excluded from the data prior to analysis: mitochondrial genes <10% and number of detected genes >200. The filtered data were normalized and scaled with batch correction using the *SCTransform* function of Seurat v4. PCA, clustering, and UMAP visualization were performed using the *RunPCA, RunUMAP*, *FindNeighbors*, and *FindClusters* functions.

### In vitro culture of tumor-infiltrating immune cells with stimulation

For in vitro stimulation of tumor-infiltrating T cells, immune cells were sorted from the tissue dissociated by MACS CD45 MicroBeads (Miltenyi Biotec, 130-052-301) and seeded at 1.25 × ${10}^{7}$ cells in each 100 mm dish. Then, T cells were activated by bead particles labeled with the CD3ε/CD28 antibody of the T Cell Activation/Expansion Kit (Miltenyi Biotec, 130-093-627). After 17 h of incubation, brefeldin A was used to block protein transport for 4 h.

### Cytokine assay of tumor macrophages

To evaluate the effects of IL-7 on the inflammatory properties of tumor macrophages, we performed a cytokine assay after in vitro IL-7 treatment of the tumor macrophages. CD11b^+^ cells were obtained using MACS CD11b MicroBeads UltraPure (Miltenyi Biotec, 130-126-725) from tumor samples from mice. Cells were seeded at 1.0 × ${10}^{6}$ cells per well in a 6 well plate and treated with recombinant mouse IL-7 protein (RnD Systems, 407-ML). After 48 h of treatment, cell supernatants were collected and analyzed using a LEGENDplex® Mouse Inflammation Panel (13-plex) (BioLegend, 740150) following the manufacturer’s instructions.

### Flow cytometry analysis

After assaying cell viability with AO/PI, cell suspensions were incubated with TrueStain FcX™ antibodies for 10 min on ice and then stained for 40 min with fluorophore-conjugated primary antibodies (Supplemental Table 2) at 4 °C in the dark. For intracellular staining of Granzyme B, Arg1, and iNOS, cells stained for surface proteins were fixed with 10% fixation solution (BioLegend, 420801) and permeabilized with Intracellular Staining Permeabilization Wash Buffer (BioLegend, 421002) at 25 °C. Thereafter, the cell suspension was incubated with the primary antibody at room temperature for 20 min in the dark. Stained samples were acquired on FACSCanto II and FACSVerse cytometers (BD science) and analyzed using FlowJo v.10.8.1.

### Immunohistochemistry

For tissue microarray analysis of 15 human cancer types, tissues were fixed in a 10% NBF solution prior to embedding in paraffin. The 7-µm thick paraffin tissue sections were deparaffinized, hydrated, and processed for heat-induced antigen retrieval using Tris-EDTA buffer (Dako, ab93684, pH 9.0, 97 °C, 15 min), and then labeled with anti-IL7R antibody. The signal was detected using the Liquid DAB+ Substrate Chromogen System (Dako, K3468). Primary antibody was omitted as a negative control (data not shown). For the individual sample staining, OCT-embedded tissues from lung cancer patients and fresh frozen tissues from colon cancer and stomach cancer patients were used. This study was approved by the Institutional Review Board of the Catholic University of Korea (IRB No. MC20SISI0108). The 7-μm thick sections were fixed with 10% NBF for 10 min at room temperature. Subsequently, the samples were washed three times with PBS, blocked for 1 h at room temperature, and then incubated with primary antibodies at 4 °C overnight. After washing three times with PBS, the samples were incubated with secondary antibody (Vector Laboratories, MP-7500), and the signal was detected using DAB substrate (TaKaRa, MK210). Finally, the sections were counterstained with hematoxylin (Abcam, ab220365) and mounted (Vector Laboratories, H-5000). Images were acquired automatically using a digital slide scanner (3DHISTECH Ltd., Pannoramic MIDI). For mouse tissue staining, HIER was performed with citrate at pH 6.0, and signal was detected using the EnVision™ Detection System (Dako, K5007).

### Immunofluorescence

Mouse tumors were frozen in OCT compound (Sakura Finetek, 4583) on 2-methyl butane. Tissues were dissected into 4–6-μm sections, fixed with cold acetone at –20 °C for 20 min, and then blocked with 10% normal donkey serum for 1 h at 25 °C. Tissue sections were then incubated overnight at 4 °C with primary antibodies. Each slide was washed three times in PBS and incubated with a fluorophore-conjugated secondary antibody for 2 h at room temperature. The nuclei were stained with DAPI, and Vectashield Antifade Mounting Medium (Vector Laboratories, H-1000-10) was used to preserve fluorescence. Stained tissues were visualized under a confocal microscope (Carl Zeiss, LSM800 with Airyscan).

## Results

### IL7R expression in early-activation stage T cells and APCs

**Fig. 1:**
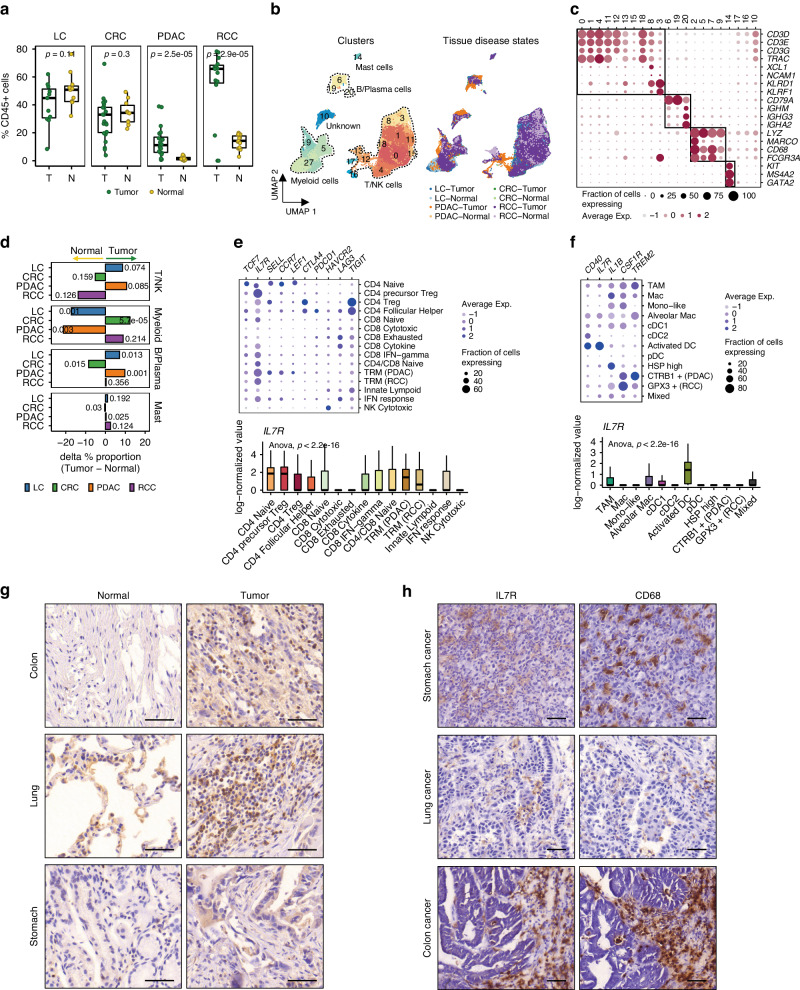
IL-7 receptor expression in myeloid and T/NK cells of primary human tumors. **a** Comparisons of CD45^+^ cell proportions between tumor and normal samples. Lung cancer (LC), colorectal cancer (CRC), pancreatic ductal adenocarcinoma (PDAC), and renal cell carcinoma (RCC). Each box represents the interquartile range (IQR, the difference between the third and first quartiles) and the median of the score, and the whiskers represent 1.5 x IQR. p, two-sided *T* test *p* value. **b** UMAP plot of 79,233 CD45^+^ cells from four human tumors, colored by clusters (left) and tissue disease states (right). **c** Dot plot of the expression of known cell lineage markers for each cell cluster. Color depicts the mean expression value scaled by z-transformation and limited to a scale from −2.5 to 2.5. Dot size depicts the fraction of cells with the gene expression value for each cluster. **d** Changes in cell lineage proportions between tumors and normal tissues. The text in the plot represents the two-sided *T*-test *p* value. **e** Mean expression of conventional immunotherapeutic target molecules for each T/NK cell subset. Dot plot of expression of selected genes (top) and box plot of *IL7R* expression for cell subsets (bottom). **f** Mean expression of conventional immunotherapeutic target molecules for each myeloid cell subset. Dot plot of the expression of selected genes (top) and box plot of *IL7R* expression for cell subset (bottom). **g** Representative image of immunostaining with IL-7 antibody in normal and tumor tissue by cancer type. **h** Immunostaining of IL7R and CD68 in colon, stomach, and lung cancer.

To identify immunotherapeutic targets, we investigated the immune cell landscape in solid tumor tissues from four scRNA-seq datasets of lung cancer [19], colorectal cancer [8], pancreatic ductal adenocarcinoma [21], and renal cell carcinoma [20], generated from at least five normal and tumor samples using comparable protocols and platforms. Following quality-filtration, we selected immune cell populations based on the expression of CD45-encoding *PTPRC* gene, and lack of *EPCAM* expression. Lung and colon cancers exhibited the highest rate of immune cell recovery in normal and tumor tissues, revealing an immune-rich TME (Fig. 1a). As previously reported, pancreatic adenocarcinoma displayed the lowest number of immune cell infiltrations, confirming a cold immune status [32]. Compared to normal tissues, renal cell carcinoma tissues underwent a drastic increase in immune cell infiltration, suggesting a robust anti-tumor immune response.

Integration of the four datasets revealed two large immune cell clusters representing myeloid and T/NK cell populations, as well as smaller B/plasma and mast cell clusters (Fig. 1b, c, and Supplementary Table 1). Comparing the fraction of cell types in normal and tumor tissues showed proportional changes in immune cells for each type of cancer (Fig. 1d). The proportion of myeloid cells in colon cancer was considerably higher than that of total B/plasma cells (*p* < 0.05). Conversely, lymphocytes such as T/NK (*p* < 0.1) and B/plasma (*p* < 0.05) were relatively abundant in lung and pancreatic cancers, whereas myeloid cells (*p* < 0.01) were comparatively low. A high proportion of macrophages is a known tissue-specific characteristic of normal lung tissues [33]. Massive infiltration of tumor-associated macrophages (TAMs) in colon cancer has been reported in multiple studies [34, 35].

In all tumor tissues, TAMs replaced resident populations such as lung alveolar macrophages, prostate CTRB1^+^ macrophages, and renal GPX3^+^ macrophages (Supplementary Fig. 1a, b, and Supplementary Table 1). Plasmacytoid dendritic cells (pDCs) were marginally elevated in lung, colon, and pancreatic cancers, whereas conventional DCs were drastically reduced in colorectal cancer (Supplementary Fig. 1c). T cells exhibited diverse gene expression profiles including naïve-like, activated, follicular helper, and regulatory CD4^+^ T cells, in addition to cytokine-rich, highly cytotoxic, and exhausted CD8^+^ effector T cells (Supplementary Fig. 1d–f, and Supplementary Table 1). Compared to normal tissues, CD4^+^ regulatory and follicular helper T cells were commonly elevated, whereas overall cytotoxic immune cell clusters (CD8 Cytotoxic, NK Cytotoxic, and Innate Lymphoid) were reduced (Supplementary Fig. 1g). In contrast, CD8^+^ T cell states were differentially regulated in each tumor type. The degree of mixing among four datasets in embeddings of CD45+ cells, Myeloid, and T/NK cells was evaluated by LISI metric [23]. Integration of the four datasets was performed appropriately to assign cell identity covering tissue-specific cell types such as lung alveolar macrophages (Supplementary Fig. 1h).

A majority of conventional immunotherapeutic target molecules, including *PDCD1* (PD-1), *CTLA4*, *HAVCR2* (TIM-3), *LAG3*, and *TIGIT*, were dominantly expressed in late-differentiation stage T cells, particularly CD4^+^ regulatory (Treg) and CD8^+^ exhausted (Tex) T cells (Fig. 1e). IL-7 receptor (IL7R), by comparison, is commonly expressed in early-differentiation stage T cells, ranging from naïve to effector/memory populations. Previous research has demonstrated that the abundance of pre-exhaustion stage T cells predicts a better response to ICIs [36–38]. These findings suggest that targeting early-activation state T cells may be an effective immunotherapeutic strategy for overcoming the low response rate of current ICIs.

Recent studies have investigated the action of IL-7 on myeloid cells in inducing arthritis or endothelium recruitment [39, 40]. The results indicate that IL7R is expressed in monocytes and macrophages. Consistent with these studies, IL7R transcripts were detected in tumor macrophages and DCs, which are professional antigen presenting cells (APCs) (Fig. 1f). To validate the protein expression of IL7R, we performed immunohistochemistry (IHC) on a tissue microarray of 15 human cancer types (Fig. 1g and Supplementary Fig. 2), which revealed positive staining of IL7R in the majority of tumor tissues. Serial section staining of IL7R and CD68 in colon-, stomach-, and lung cancers confirmed strong expression of IL7R in the macrophage compartment (Fig. 1h). These data suggest that IL7R signaling by recombinant IL-7 (rIL7) activates T cells and APCs, facilitating simultaneous innate and adaptive immune responses.

### Mouse transplant model mimics the TME of human solid tumors

Defining the altered immune landscape in primary human tumors would expedite the discovery of therapeutic targets for inhibiting immune suppression and stimulating anti-tumor immunity. Next, we required a model system that mimics the human TME and can be manipulated to promptly assess the potential of novel targets. Syngeneic tumor transplant models in mice have been adopted in order to evaluate the potential and efficacy of immune checkpoint inhibitors [41, 42]. scRNA-seq has further been used to demonstrate the effects of immune checkpoint inhibitors on transcriptional-state immune cell dynamics [43].

**Fig. 2:**
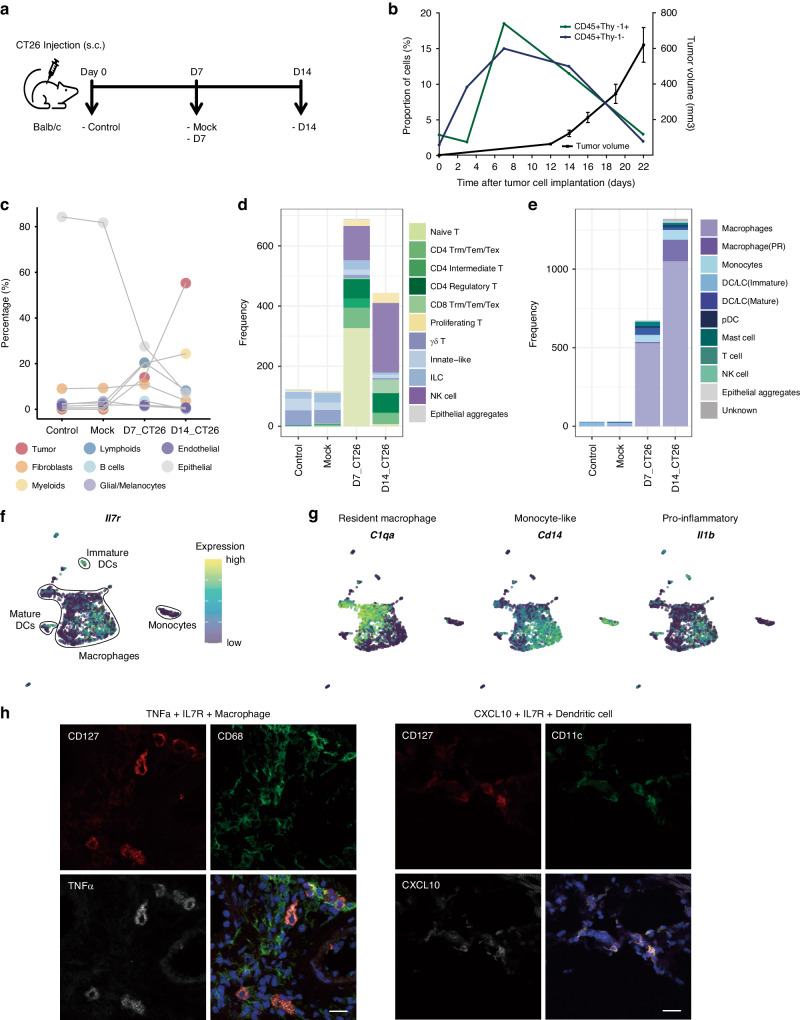
Immune cell dynamics in a syngeneic mouse transplant model. **a** A tumor transplant model was established by subcutaneous inoculation of the CT26 cell line into BALB/c mice. Control and mock samples were injected with PBS. **b** Growth curve of CT26 tumors in syngeneic BALB/c mice. The left y-axis indicates percentage of CD45^+^ Thy-1^+^ and CD45^+^ Thy-1^-^ cells in single cell suspension from tumor tissue. Tumor volume (right y-axis) was measured by Mean ± SEM (*n* = 10). **c** Cell type percentage graph illustrated an increase in lymphoid and myeloid cells during tumor growth. Frequency bar plots for subsets of lymphoid (**d**) or myeloid (**e**) cell types. Colors represent each subset. **f** UMAP plot of 2057 myeloid cells and their expression level of *Il7r* gene. The dot colors represent gene expression levels. **g** UMAP plot of representative macrophage feature genes including *C1qa* (resident macrophage), *Cd14* (monocyte-like), and *Il1b* (pro-inflammatory) in myeloid cells. As shown in (**f**), the dot colors represent gene expression levels. **h** Immunofluorescence staining of CD127 (IL7R), CD68, and TNFα (left) or CD127, CD11c, and CXCL10 (right) in mouse tumors. Images were presented individually or were merged together (right bottom). Scale bar, 20 µm.

We adopted the CT26 colon carcinoma cell line model [44] and examined the cellular dynamics during tumor growth in BALB/c recipients to determine the degree to which the mouse model resembles the human TME (Fig. 2a, b). Histological staining and flow cytometry demonstrated that as the tumor grew in the subcutaneous layer of the skin, immune cell infiltration increased first by myeloid cells and then by T cells (Fig. 2b and Supplementary Fig. 3). Immune cell proportions began to decline on day 14 post-transplantation, and only a small fraction of immune cells remained on day 22 when extensive tumor necrosis occurred. We applied scRNA-seq to the skin and tumor mass at days 0, 7, and 14 post-transplantation to depict how tumor growth replaced or altered resident cells in normal. Subsequently, a total of 19,280 cells were analyzed with an average of 4041 unique molecular identifiers (UMIs) and 18,310 genes for four groups: control at day 0 (control), mock-injection control at day 7 (mock), an early phase of tumor-transplant at day 7 (D7), and later phase of tumor-transplant at day 14 (D14).

In the control and mock transplant groups, we recovered relatively few immune cells from the skin layers. Numerous myeloid and lymphoid cells had infiltrated the tumor mass by days 7 and 14, respectively, following tumor transplantation (Fig. 2c and Supplementary Fig. 4). On day 7, tumors comprised 15% of the total cells, while myeloid and lymphoid cells accounted for 22% and 20%, respectively. Owing to rapid tumor growth, tumor cells constituted over 50% of the total cells by day 14, with myeloid and lymphoid cells accounting for 25% and 10%, respectively. These results indicate that myeloid cells expand earlier and remain longer in the TME than T cells.

Infiltrating immune cells undergo striking phenotypic shifts and proportional alterations during tumor growth. In the lymphoid compartment, naïve-like CD4^+^ and CD8^+^ T cells prevailing in the D7 transplant are gradually activated and differentiated into effector or memory T cells (Fig. 2d and Supplementary Fig. 5a–e). Exhausted CD8^+^ T cells, considered to be terminal-state effector T cells post-chronic stimulation, were predominantly found in the D14 transplant. Notably, the regulatory T cell cluster emerged early in D7 and persisted until the D14 transplant phase. In the myeloid compartment, monocytes and monocyte-like macrophages were discovered in both D7 and D14 transplants, depicting continuous recruitment of blood- or bone-marrow-derived populations (Fig. 2e and Supplementary Fig. 6a–c). On day 14, proliferative and differentiated macrophages were highly enriched with more anti-inflammatory macrophages than on day 7 (Fig. 2e and Supplementary Fig. 6d).

Comparison of cluster-wise annotations of human and mouse data with each other confirmed the detection of lineage-level subsets such as naïve T, CD8 + T, CD4 + T, CD4+ Treg, macrophage, DC, and pDC in both human and mouse data (Supplementary Figs. 5e and 6e). As is already well known, however, a few marker genes have different expression patterns due to differences in species. For example, orthologs of *KLRF1* and *CD1C* are absent in mouse and *Siglec-H* in human. Expression of *NCAM1* (for NK), *FCN1* (for monocyte), *MARCO* (for resident macrophage), *CLEC4B1* and *IL3RA* (for pDC) was high only in human cells. There were also some differences in clustering between two species. The human data had tissue type-specific subclusters such as CD4+ Tfh, Trm from PDAC and RCC, and alveolar macrophages that could not be included in the mouse model. Monocytes and Tγδ were clustered in the mouse data, but not in the human data. These types of cells may be spread out in other clusters with similar transcriptomic profiles and could be collected by additional analysis, which is supported by the fact that mast cell clusters appeared upon total cell clustering in the human data but upon myeloid cell clustering in the mouse data (Fig. 1b and Supplementary Fig. 6e). Overall, we proposed that the TME of solid human tumors comprising exhausted CD8^+^ T cells and anti-inflammatory resident macrophages was reconstructed between days 7 and 14 post-transplantation.

### rIL7 inhibits tumor growth by stimulating myeloid and T cell

To determine the anti-tumor efficacy of rIL7, we first analyzed IL7R expression in the mouse tumor transplant model. Expression of *Il7r* transcripts, particularly in early-activation state T cells, as well as in subpopulations of macrophages and DCs, were consistent with that of human tumors (Fig. 2f and Supplementary Fig. 7a–d). In T cells, a positive correlation was observed between the expression levels of *Il7r* and *Ramp3* (Supplementary Fig. 7b). The expression of receptor activity-modifying protein 3 (RAMP3) on unstimulated human T cells is known to be downregulated by phytohemagglutinin (PHA) stimulation [45]. In myeloid compartments, Il7r-expressing macrophages highly expressed inflammatory cytokine (*Il1b*) and monocytic gene (*Cd14*) but not the resident macrophage gene (*C1q*) (Fig. 2g). The *Il7r* expression correlation analysis revealed that macrophages with high *Il7r* levels were pro-inflammatory (*Csf2rb, Cxcl2*, and *Clec4e*) (Supplementary Fig. 7d) [27, 46]. In mature DCs, the expression of *Il1b* and antigen-presenting molecules (MHC II and *Cd74*) exhibited positive correlation with *Il7r* expression (Supplementary Fig. 7d). The correlation between *Il7r* expression and antigen-presenting molecule expression can be explained by the effect of IL-7 signaling on DC maturation. Vogt et al. reported that IL-7 signaling is required for DC development and IL7R expression is maintained only in a subset of mature DCs, including migratory DCs [47]. Immunofluorescence staining of tumor tissues further confirmed the cell surface expression of IL7R (CD127) on macrophages and DCs, and the presence of pro-inflammatory CD127^+^ macrophages and DCs (Fig. 2h). Consequently, we anticipated that rIL7 therapy would stimulate the early-differentiation state T cells and pro-inflammatory APCs.

**Fig. 3:**
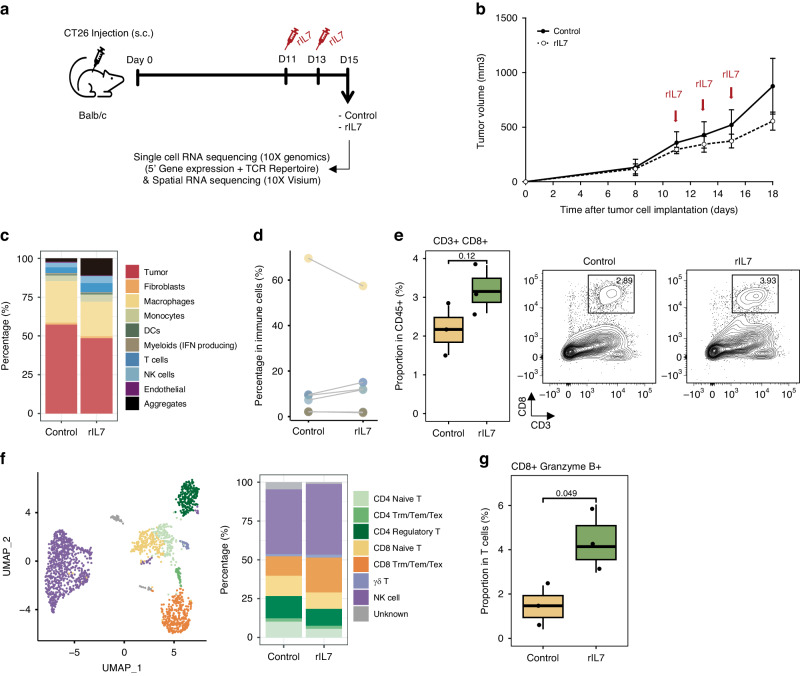
IL-7 treatment inhibits tumor growth and co-stimulates inflammatory macrophages and CD8^+^ effector T cells. **a** A tumor transplant model for rIL7 treatment was developed by subcutaneous inoculation of the CT26 cell line into BALB/c mice. Recombinant IL-7 (rIL7 group) or PBS (control group) was intraperitoneally injected twice at days 11 and 13 post-transplant. Tumor tissues were collected on day 15. **b** Growth curve of CT26 tumors in syngeneic BALB/c mice after rIL7 treatment. Tumor volume was measured by Mean ± SEM (*n* = 10 for control; *n* = 5 for rIL7). **c** Percentage bar plot of cell types in each sample. Colors denote the cell types. **d** Comparison of the proportion of immune cell types such as macrophages, monocytes, DCs, IFN producing myeloid, NK, and T cells among the total of 8,560 immune cells. Colors represent each cell type as shown in (**c**). **e** Flow cytometry analysis illustrates an increase in CD8^+^ T cell (CD3^+^ CD8^+^) proportion after rIL7 treatment. Tumor specimens were collected 15 days after tumor implantation in the CT26 syngeneic mouse model (*n* = 3). **f** UMAP and subset proportion bar plots of 1818 T/NK cells. Colors represent each T/NK subset type. **g** Boxplot of percentage of CD8^+^ GranzymeB^+^ cells in the CD45^+^ CD3^+^ T cell fraction (*n* = 3), with a *p* value for the two-sided *T* test.

**Fig. 4:**
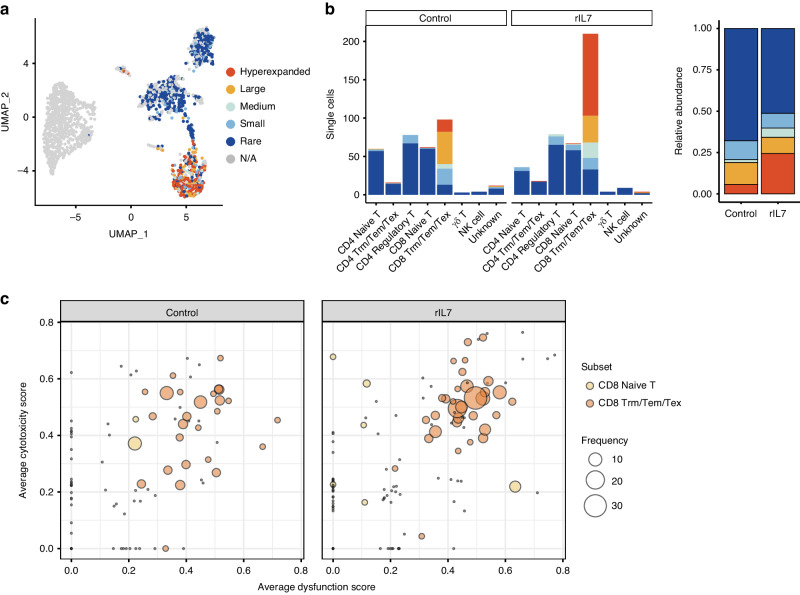
IL-7 stimulates expansion of CD8^+^ effector T cells with proliferative capacity and prolongs the maintenance of effector function. **a** UMAP plot of T/NK cells colored by T cell clonotype. Single=1, Small=2, Medium=3, Large ≤5, and Hyperexpanded >5 copies. **b** Frequency of clonotype in each subset (left) and their relative abundance (right) in each sample. Colors represent clonotypes as defined in (**a**). **c** Bubble plots of CD8^+^ T cells represent the expansion of effector CD8^+^ T cells in the rIL7 group. Each bubble represents a clone and is colored by subset and sized by clonal frequency. The x- and y-axes depict dysfunctional and cytotoxicity scores, respectively.

Based on the immune cell dynamics, we administered rIL7 on day 11 post-transplantation to detect anti-tumor activity in the TME enriched in exhausted T cells and anti-inflammatory macrophages (Fig. 3a). scRNA-seq was performed 4 days post-rIL7 administration and 15 days post-transplantation to characterize early changes in the composition and phenotype of immune cell populations. Multiple rIL7 injections at 48-h intervals inhibited tumor growth (Fig. 3b). Following rIL7 treatment, we observed a slight reduction in tumor size and tumor cell proportion, as well as an increase in T and NK immune cells (Fig. 3b–d and Supplementary Fig. 8a–d). The increase in T cells by rIL7 was confirmed by flow cytometry analysis in a separate experiment (Fig. 3e). Further sub-clustering of T/NK lymphoid populations revealed an increase in CD8^+^ effector T cells in the rIL7 treatment group, including resident memory, effector memory, and exhausted gene expression phenotypes (Trm/Tem/Tex) (Fig. 3f and Supplementary Fig. 9a, b). T cells in the rIL7 group displayed higher cytotoxic and exhausted scores than those in the control group (Supplementary Fig. 9c). An increase in CD8+ cytotoxic T cells expressing granzyme B in IL7-treated mice was re-confirmed by independent experiments (Fig. 3g and Supplementary Fig. 9d). TCR sequence analysis demonstrated clonal expansion of the CD8^+^ Trm/Tem/Tex cluster in response to rIL7 treatment (Fig. 4a, b), with high cytotoxic gene expression (Fig. 4c). Although we expected a substantial overlap of tumor-specific clones between individual mice, there was no clonal overlap between control and IL7-treated mice (Supplementary Fig. 9e). This may be explained by the stochastic nature of TCR generation (potentially 10^15^–10^20^ clonal diversity) and the relatively small size of the T cell repertoire (~10^7^ clones) in a mouse [48]. Instead, IL-7-induced PD-1^+^ effector T cell expansion was confirmed by flow cytometry in vitro (Supplementary Fig. 9f).

Monocyte or macrophage clusters constituting the control (clusters 9 and 14; clusters 3 and 6) or rIL7 groups (cluster 5; cluster 11) were imparted by myeloid cell populations (Supplementary Fig. 10a–c). Differential expression gene analysis (adjusted *p* < 0.05, log2(fold-change) >0.25) revealed downregulation of TAM-associated gene (*Spp1*) [7] and upregulation of pro-inflammatory genes (*Il1b* and *Csf3r*) in the rIL7 group (Supplementary Fig. 10d). Flow cytometry analysis confirmed that macrophages (CD45^+^ F4/80^+^) from the rIL7 treatment group manifested Arg1^low^iNOS^high^ status compared with those from the control group (Supplementary Fig. 10e, f); iNOS and Arg1 mediate proinflammatory and anti-inflammatory macrophage functions, respectively [49]. We isolated myeloid cells (CD11b^+^) from the control transplant and incubated them with rIL7 in vitro to assess whether IL-7 is directly involved in the pro-inflammatory conversion. Myeloid cells subjected to rIL7 treatment produced elevated levels of inflammatory cytokines (Supplementary Fig. 10g). Hence, the pro-inflammatory phenotype almost certainly promotes an immunogenic microenvironment and aids tumor regression.

### rIL7 elevates macrophage–CD8^+^ effector T cell interaction

In addition to the immunogenic transition of myeloid and lymphoid immune cells, rIL7 treatment promoted the formation of multicellular aggregates (clusters 14, 15, 21, and 22) that expressed multiple cell type lineage markers, including CT26 tumor markers (*Rpl39l* and *Baiap2l1*) and macrophage markers (*H2-Aa* and *Cd68*) (Supplementary Fig. 8c). These multicellular aggregates may represent doublet formation or the fusion of tumor and macrophages [50, 51]. Tumacrophages are phagocytosis-generated fusions of apoptotic tumor cells and macrophages. Inter-cluster correlations displayed two types of multicellular aggregates: tumacrophage-like (cluster 14) and immune-like (cluster 15) (Supplementary Fig. 8d). The increase in tumacrophage-like aggregates in the rIL7 treatment group indicates a macrophage-tumor cell interaction or apoptosis-dependent phagocytosis. Furthermore, immune-like aggregates may appear due to elevated APC-T cell interactions.

**Fig. 5:**
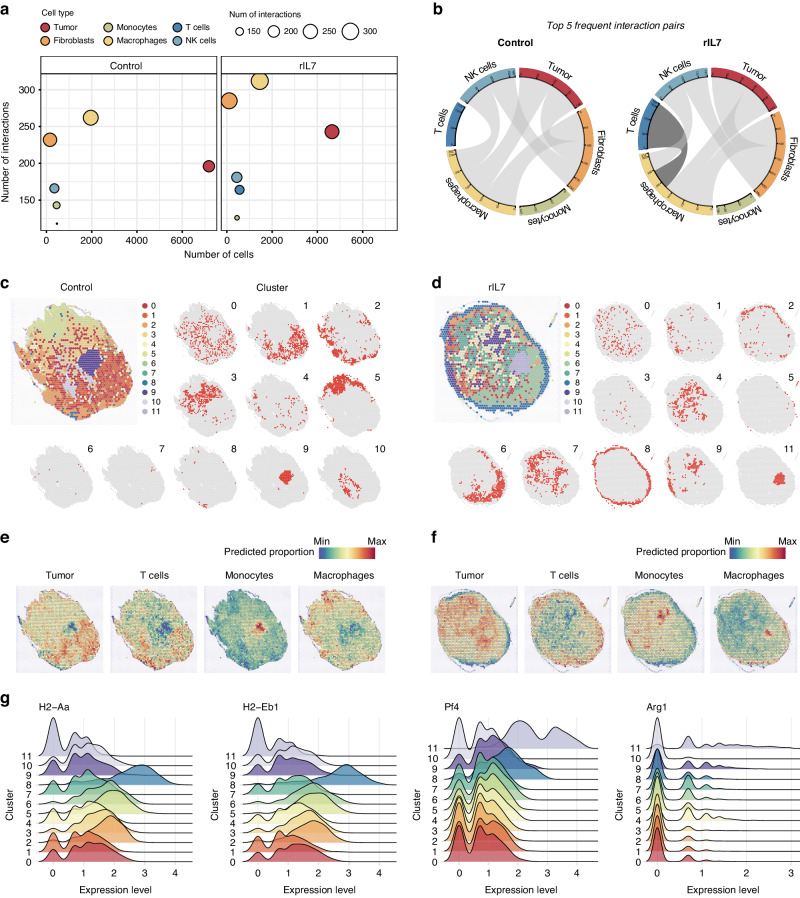
Enhancement of the macrophage and T cell interactions by IL-7 treatment. **a** Bubble plot displays an increase in interactions in macrophages, fibroblasts, and T cells after rIL7 treatment. Each bubble represents a major cell type and is sized according to the number of interactions occurring within that cell. The x-axis and y-axis indicate the number of cells and number of interactions, respectively. **b** Circos plot of the top 5 frequently interacting cell-type pairs. Gray ribbons link interactive cell types. The black ribbon in the rIL7 group (right) indicates distinction from the control group (left). Each band of the Circos plot is drawn per cell type, and the length of the band is determined by the total number of interactions in that cell. Tissue images for control (**c**) and rIL7 (**d**) samples depicted as an overlay of the spot cluster annotation. Tissue images for control (**e**) and rIL7 (**f**) samples overpainted by the predicted proportion of tumor, T cells, monocytes, and macrophages. **g** Expression levels of APC related genes such as MHC class II (*H2-Aa* and *H2-Eb1*), *Pf4* (pro-inflammatory), and *Arg1* (anti-inflammatory) in each spot cluster.

To depict alterations in intercellular communication induced by rIL7 treatment, we initially employed CellphoneDB [29], a statistical method for inferring ligand-receptor interactions from gene expression data. Overall, the ligand-receptor interactions predicted for the rIL7 treatment group were more frequent compared to the control group, validating rIL7-stimulated intercellular communications in the TME (Fig. 5a); among them, the macrophage-T cell pair emerged as a frequently interacting pair in the rIL7 group (Fig. 5b).

Spatial transcriptome profiling further supported the reinforcement of macrophage-T cell interactions, allowing gene expression patterns to be mapped over tissue location [52]. Prediction of the cell type proportion correlation elucidated that rIL7 promotes the transition from a microenvironment rich in tumor-T interactions to one rich in APC-T and tumor-monocyte interactions (Supplementary Fig. 11a). Indeed, tumor-T (C1), APC-T (C8), and tumor-monocyte (C4) colocalization was enhanced in the control or rIL7 samples as predicted (C1, control-enriched; C4 and C8, IL-7-enriched) (Fig. 5c–f and Supplementary Fig, 11b–d). The C8 cluster prevalent on the tumor periphery of rIL7-treated tumors exhibited the highest T cell, macrophage, and DC signatures and expressed higher levels of MHC class II (*H2-Aa* and *H2-Eb1*) and *Pf4* genes rather than *Arg1* genes (Fig. 5g). These results signify the colocalization of T cells and pro-inflammatory APCs, suggesting that rIL7 treatment promotes their interactions.

**Fig. 6:**
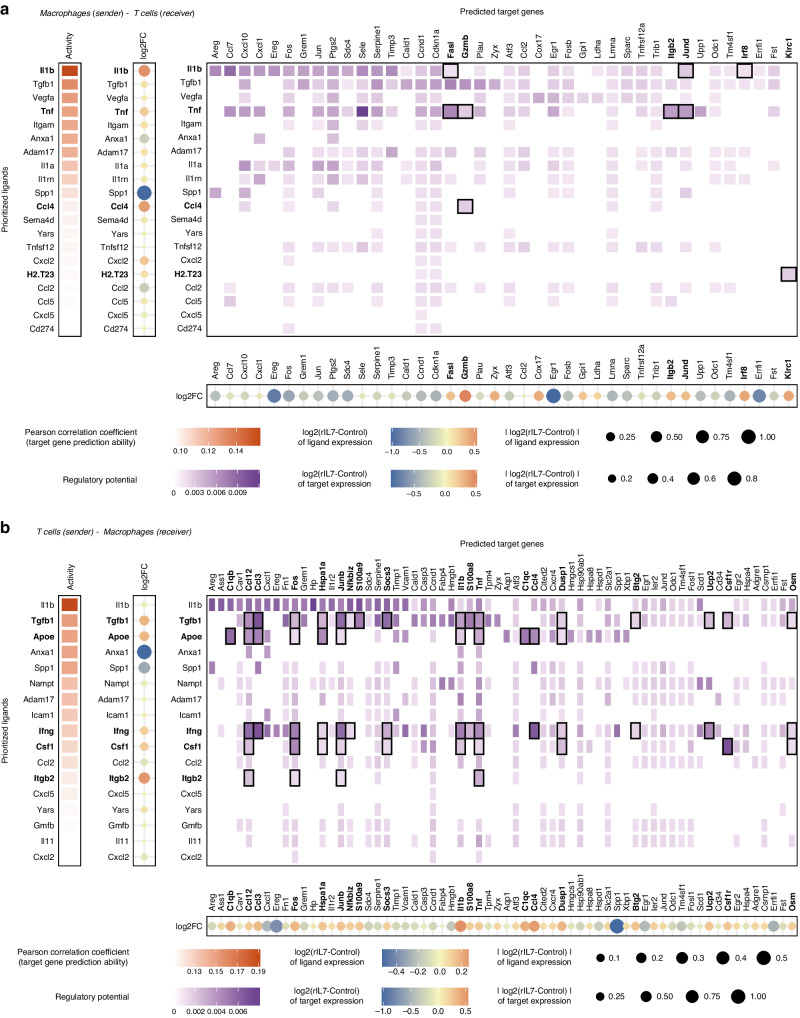
IL-7 treatment induces anti-tumoral interactions between macrophages and T cells. NicheNet analysis of cellular communication between macrophages and T cells depicting potential alteration by rIL7 treatment. **a** Macrophages express high levels of inflammatory cytokines such as *Il1b*, *Tnf*, and *Ccl4* after rIL7 treatment, which stimulates overexpression of *Fasl*, *Gzmb*, *Itgb2*, *Jund*, and *Irf8* in T cells. **b** T cells express elevated levels of inflammatory genes such as *Itgb2, Tgfb1, Csf1*, and *Ifng*, which induces overexpression of *Il1b, Ccl4, Fos, Ccl3, Junb, Csf1r, Tnf*, and *Ccl12* in macrophages. Black box in heatmap indicates a remarkable link that meets log2-foldchange of ligand expression >0.1, log2-foldchange of target expression >0.2, and regulatory potential >0.

Inference of ligand-receptor pairs between macrophages and T cells depicted communication in the rIL7 group. The impact of altered cellular communications between macrophages and T cells was demonstrated by NicheNet analysis [30], a method linking ligands to target gene expression. In the rIL7 treatment group, macrophages displayed elevated expression of inflammatory cytokines such as *Il1b*, *Tnf*, and *Ccl4*, which likely influenced the expression of *Fasl*, *Gzmb*, *Itgb2*, *Jund*, and *Irf8* in the T cells (Fig. 6a). Fas ligand (*Fasl*) and granzyme B (*Gzmb*) are the principal cytotoxic mediators of T cell-mediated apoptosis [53, 54]. Integrin beta 2 (*Itgb2*) is a subunit of lymphocyte function-associated antigen 1 (LFA-1), a binding molecule required for interaction with APCs. These APC-T interactions are manifested through LFA-1-mediated T cell activation and migration [55]. The role of JunD (*Jund*) in promoting IL-7-induced CD8^+^ T cell proliferation has been previously enunciated [56]. Interferon regulatory factor 8 (*Irf8*), a transcription factor expressed in CD8^+^ T cells, is capable of inducing the differentiation of naive CD8^+^ T cells into effector cells [57]. T cells in the rIL7 treatment group expressed immunostimulatory genes, including *Itgb2, Csf1*, and *Ifng*, to a greater extent, and induced the expression of pro-inflammatory genes, including *Il1b, Ccl4, Fos, Ccl3, Junb, Csf1r, Tnf*, and *Ccl12*, in macrophages (Fig. 6b). These results indicate that the construction of an anti-tumor environment is mediated by T cell–macrophage interactions.

## Discussion

Utilizing single-cell transcriptome technologies, we have deepened our understanding of the anti-tumor effects of IL-7 and discovered new strategies for employing IL-7 in anti-cancer therapy. Previous studies have focused on T cells to explain the anti-cancer function of IL-7. Adjuvant IL-7 enhanced T cell effector function by increasing IL-6 production and T helper type 17 cell differentiation, resulting in improved anti-tumor responses and overall survival [58]. Long-acting rhIL-7-hyFc increased the number of central memory and effector memory CD8^+^ T cells enhancing IFNγ production and survival [59]. Ex vivo tumor cultures and patient-derived xenograft models demonstrated that human IL-7 upregulated pro-inflammatory cytokines and activated infiltrating CD4^+^ and CD8^+^ T cells in a dose-dependent manner [60]. Pachynski et al. reported that rhIL-7 elevates the expression of lymphocytes, including CD4^+^ T, CD8^+^ T, and CD56^bright^ NK cells, in metastatic castration-resistant prostate cancer (mCRPC) patients treated with sipuleucel-T, thereby enhancing their immunity [61]. In this paper, we replicated former results using scRNA-seq and scTCR-seq data. The pleiotropic functions of IL-7 in inducing T cell proliferation and activation are validated by an overall increase in T cell numbers and the clonal expansion of effector T cells. Although not pursued in our study, tumor antigens can be addressed using effector T cell clones expanded at the tumor site by IL-7 treatment.

IL-7 was reported to activate macrophages under inflammatory conditions [39, 40], elucidating its potential for simultaneously activating lymphoid and myeloid compartments. Our study demonstrated that IL-7 affects monocyte recruitment to tumor sites and the induction of pro-inflammatory APCs. Furthermore, we discovered a synergistic effect of rIL7 treatment on APCs and T cells using scRNA-seq network analysis and spatial transcriptome profiling. Through robust APC-T cell interactions, IL-7 was predicted to induce cytotoxic CD8^+^ T cell expansion. A dominant expression of *Il7r* was observed in *Il1b*-expressing macrophages even in the absence of exogenous IL-7, suggesting that inflammatory macrophages may have been induced due to the expansion of pre-existing pro-inflammatory macrophages. Alternatively, IL-7 treatment may have triggered the differentiation of recruited monocytes or the phenotypic conversion of the resident macrophages into pro-inflammatory macrophages.

Syngeneic mouse tumor transplant models have limitations in mimicking drug effects seen in human cancer patients. One reason for this could be that many studies started drug treatment within 1 week after tumor cell injection, when the TME differs considerably from that of human. Our time-course analysis (Fig. 2a–e) revealed that the day 14 but not the day 7 post-transplantation replicated the typical tumor immune microenvironment, comprising exhausted T cells and resident macrophages. To better simulate the microenvironment of human solid tumors and test drug efficacy, testing time points in mouse syngeneic transplant models should be set at least 7 days after transplantation. Applying these results to further drug treatment study, we treated mice with rIL7 on days 11 and 13 after transplantation. Treating IL7 at a different time than we did may lead to different effects.

Nevertheless, immunologic differences in species and tissue sites as well as the natural tumor origin may still be important factors determining therapeutic outcome. Parallel comparison between human and mouse subclusters presented the similarities and differences of the human and mouse model (Supplementary Figs. 5e and 6e). Although major subsets were consistently observed in both data, tissue-specific cell clusters or differences in some marker expression represented the differences between the two data. Meanwhile, cluster-wise annotation can add to the difficulty of comparing the results from two data. Cluster-wise annotation is a general and efficient strategy for cell type identification, especially in scRNA-seq, but it has drawbacks. This method uses the average expression level of the cells to profile each cluster, so the major population of the cluster determines the annotation. In the case of cells that cannot form a cluster for some reason (e.g., low cell number, similar transcriptome profile to cells in other clusters, etc.) may be masked. Because of these limitations, constructing appropriate model system and interpreting the results carefully while avoiding leaps are needed.

However, it is important to note that IL-7/IL-7R-mediated signaling may exert pro-tumor functions [16]. Previous studies depict that high levels of IL-7 and IL-7R expression is associated with lymph node metastasis, poor survival, and poor prognosis in breast, lung, and prostate cancers. Induction of cancer cell migration, epithelial-mesenchymal transition, and tumor growth by IL-7 has been demonstrated by in vitro and in vivo studies. We also observed the expression of IL-7 and IL-7R in various mouse and human cell types and tumor tissues, which suggests that targeting the IL-7/IL-7R pathway presents both an opportunity and a risk for cancer patients. To avoid pro-tumor potential and maximize the anti-cancer effect of IL-7 treatment, refined patient selection is highly recommended. Furthermore, the impact of increasing cytotoxic CD8^+^ T cells needs to be investigated in depth. Despite the expected anti-tumor role of cytotoxic T cells, the increase in infiltrated T cells may contribute to the composition of the pro-tumoral environment through exhaustion by the tumor environment.

In this study, we demonstrated high-resolution profiling of immune cells and their cellular networks within the tumor through the integrated use of multiple single-cell genomic technologies. Based on these results, we propose that rIL7 treatment is an immunotherapeutic strategy that influences both APCs and T cells and enhances the interactions between them. Moreover, we hypothesize that IL-7 treatment may complement current ICIs by mobilizing naïve T cells into an early-activation state. Sophisticated profiling using single-cell technologies could be crucial to achieving the precise success criteria of IL-7 monotherapy as well as adjuvant therapy.

### Supplementary information

Supplementary Figures

Supplementary Table 1

Supplementary Table 2

## Data Availability

Public human scRNA-seq data are available in the NCBI Gene Expression Omnibus (GEO) or Genome Sequence Archive (GSA) databases: GSE131907, GSE132465, and CRA001160. The scRNA-seq data from renal cell carcinoma were obtained from Data S1 of the original paper. The scRNA-seq and spatial transcriptome data generated by this study are accessible through the NCBI GEO database (GSE205307).
