# Supplementary material for: Single-cell RNA sequencing reveals myeloid and T cell co-stimulation mediated by IL-7 anti-cancer immunotherapy
Source: Br J Cancer. 2024 Feb 29;130(8):1388–401. doi: 10.1038/s41416-024-02617-7 (PMC11014989; doi:10.1038/s41416-024-02617-7)
Supplement: Supplementary file 1 — Supplementary Figures [file 41416_2024_2617_MOESM1_ESM.pdf]

Supplementary Figure 1

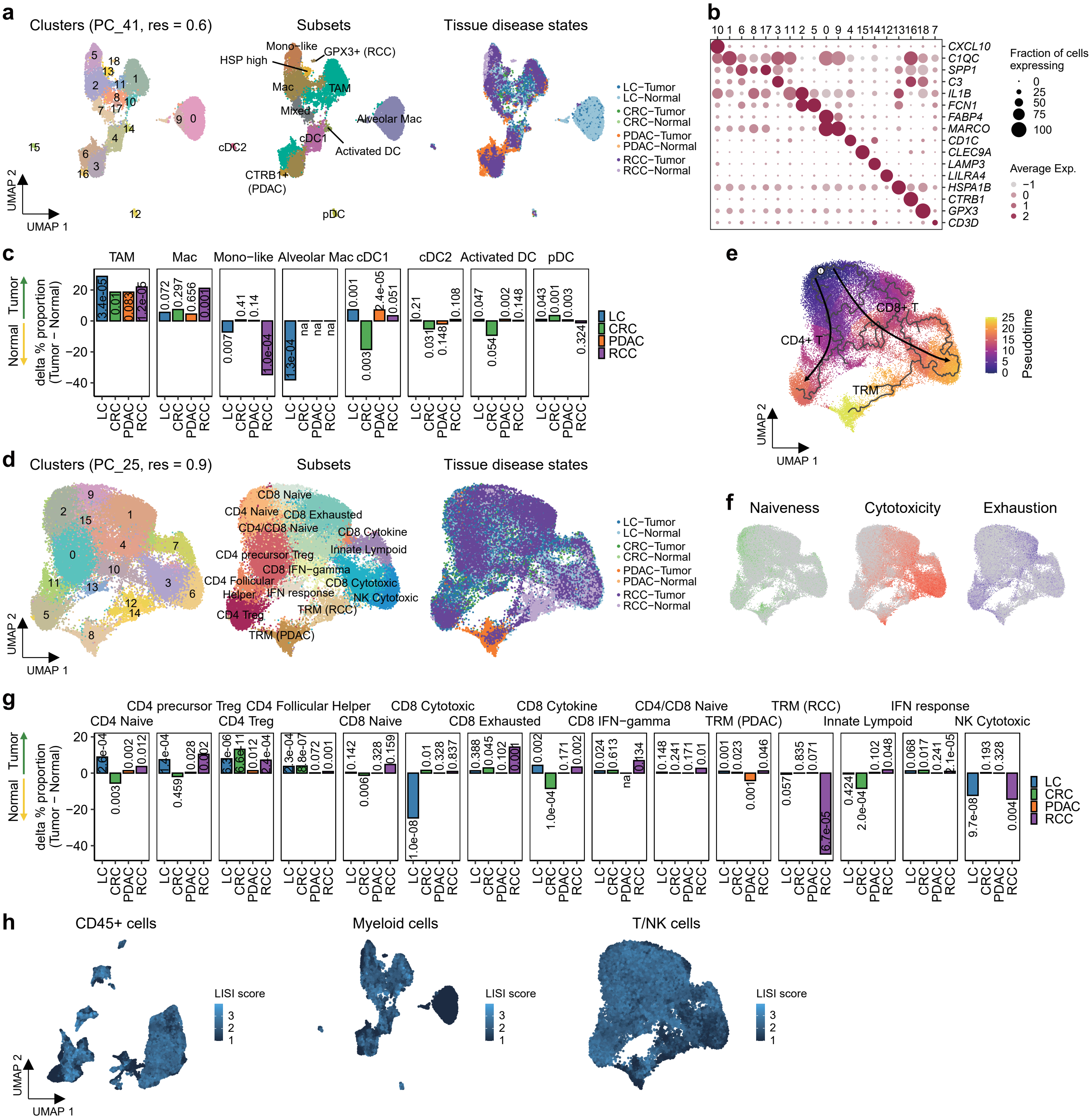

Supplementary Fig 1. Sub-clustering of myeloid and T NK cell populations from human scRNA-seq data. a, UMAP plot of 18,372 myeloid cells from four human tumors, colored by clusters, cell subsets, and tissue disease states as indicated. **b,** Dot plot of expression of selected markers for each myeloid cell cluster. Color depicts mean expression value scaled by z-transformation and limited to a scale from −2.5 to 2.5. Dot size depicts the fraction of cells with the gene expression value for each cluster. **c,** Changes in myeloid cell subset proportions between tumors and normal tissues. The text in the plot represents the two-sided T-test p-value. **d,** UMAP plot of 52,034 T/NK cells from four human tumors, colored by clusters, cell subsets, and tissue disease states as indicated. **e and f,** UMAP plot of T/NK cells colored by pseudotime estimated by Monocle 3 (e) and T cell functionality scores (f). **g,** Changes in T/NK cell subset proportions between tumors and normal tissues. The text in the plot represents the two-sided T-test p-value. **h,** UMAP plot of CD45+, myeloid, and T/NK cells from four human tumors, colored by LISI score.

Supplementary Figure 2

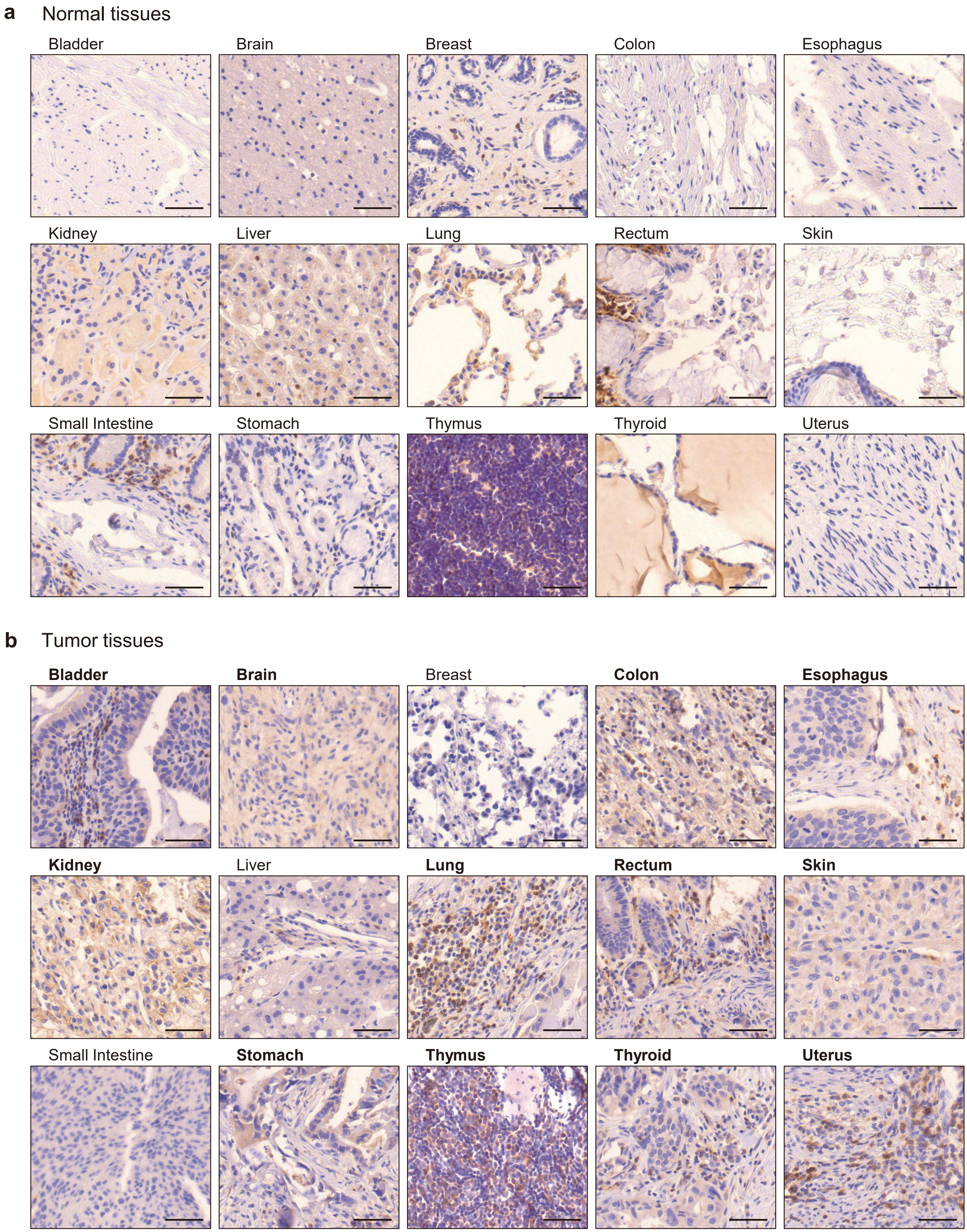

Supplementary Fig 2. IL7R expression in 15 human cancer types using immunohistochemistry array. Immunohistochemistry staining of IL7R in normal (a) and tumor (b) tissues of 15 cancer types. Scale bar, 50 µm.

Supplementary Figure 3

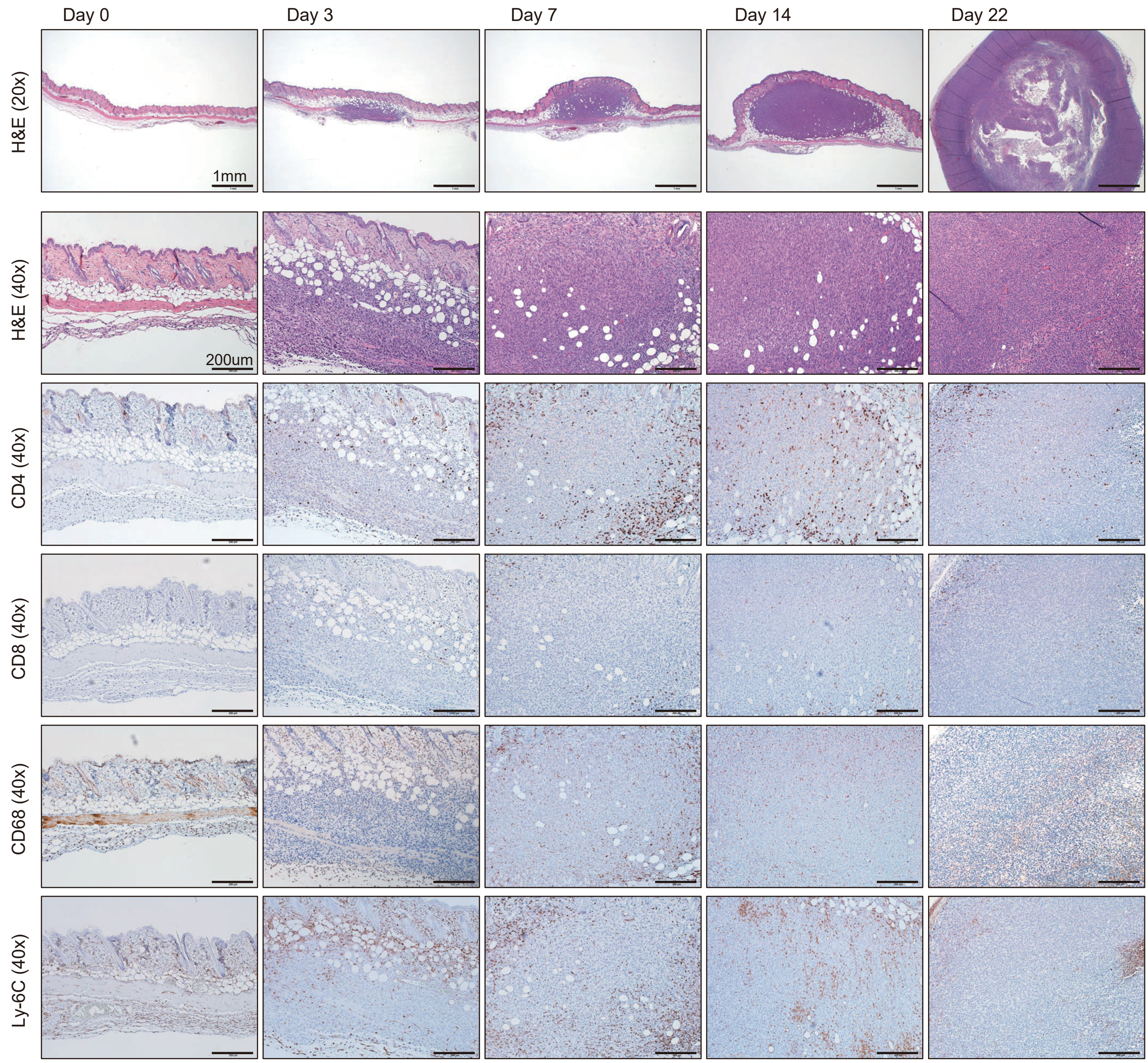

Supplementary Fig 3. Immunohistochemistry for immune cell invasion in a syngeneic mouse model. Histochemistry images of immune cell invasion in the CT26 syngeneic tumor model. Tumor tissues adjacent to skin were stained with hematoxylin & eosin (H&E) and immune cell marker panel (CD4 for CD4+ T, CD8 for CD8+ T, CD68 for macrophage, and Ly6C for monocyte). Scale bars of 20x images are 1mm (upper) and that of 40x images are 200 μm (lower).

Supplementary Figure 4

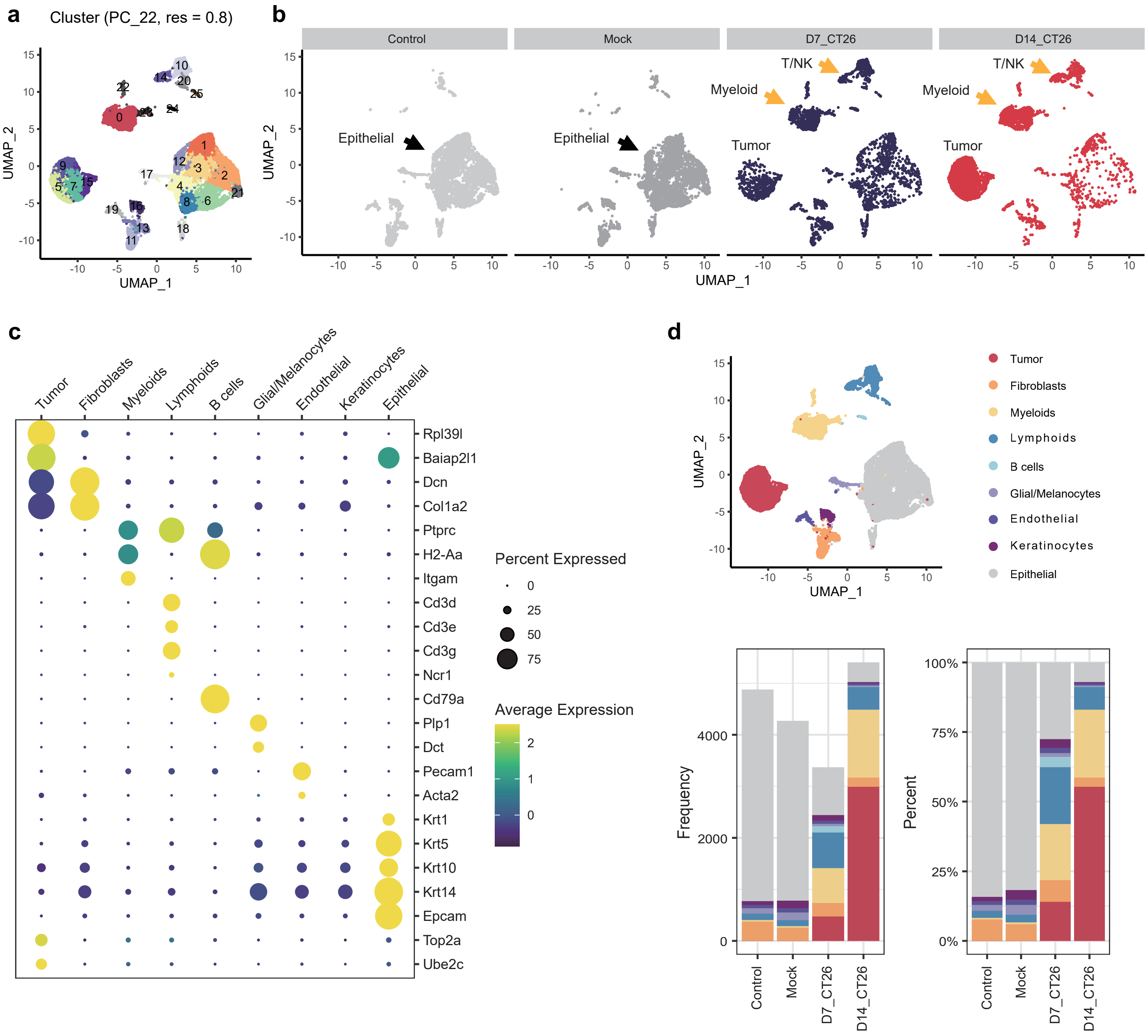

Supplementary Fig 4. scRNAseq data of mouse tumor model during tumor growth. a, UMAP plot of 19,280 cells from control at day 0 (Control), mock-injection control at day 7 (Mock), CT26 tumor-transplant at day 7 (D7), and CT26 tumor-transplant at day 14 (D14). Each dot represents a cell and colored by cluster. **b,** UMAP plots separated by sample. Cell types enriched in the sample were annotated by arrows. **c,** Dot plot of expression of known cell lineage markers for each cell type. Color depicts mean expression value and size depicts the fraction of cells with the gene expression. **d,** UMAP plot (upper) and frequency or percentage bar plots (lower). Color depicts a cell type.

Supplementary Figure 5

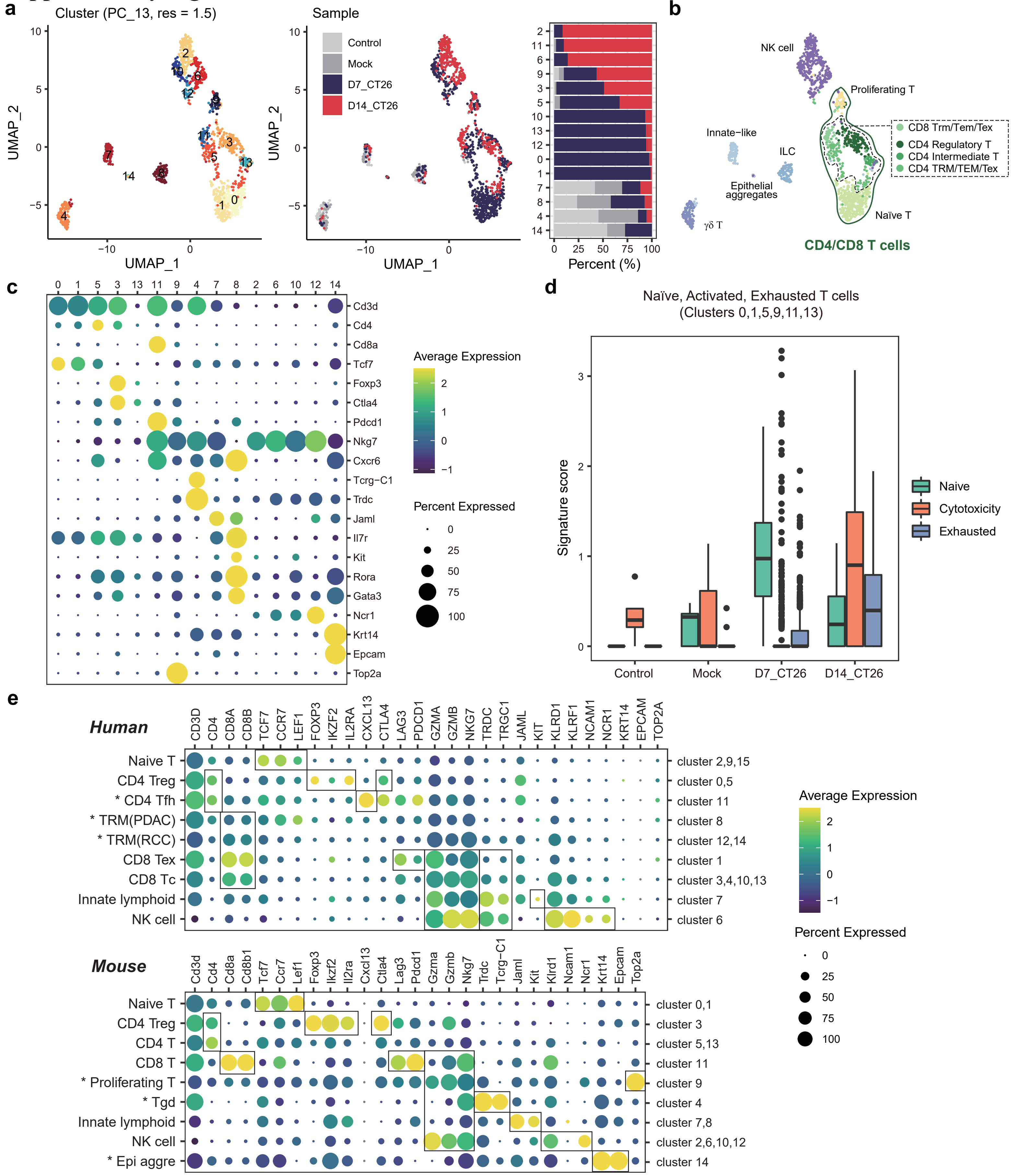

Supplementary Fig 5. Analysis of lymphoid subset using scRNA-seq during tumor growth. a, UMAP plots of lymphoid cells (left) and corresponding bar plot of sample proportions in each cluster (right). UMAP plots were colored by cluster or sample as indicated. **b,** UMAP plot colored with subsets of lymphoid cells. **c,** Dot plot of expression of known cell subset markers in each cluster. Color depicts mean expression value and size depicts the fraction of cells with the gene expression. **d,** Boxplot of T cell functionality scores in each sample. **e,** Dot plots of human and mouse data for known marker genes of each lymphoid subset. Box indicates subset-specific marker expression and asterisk (*) indicates a subcluster detected in only one species.

Supplementary Figure 6

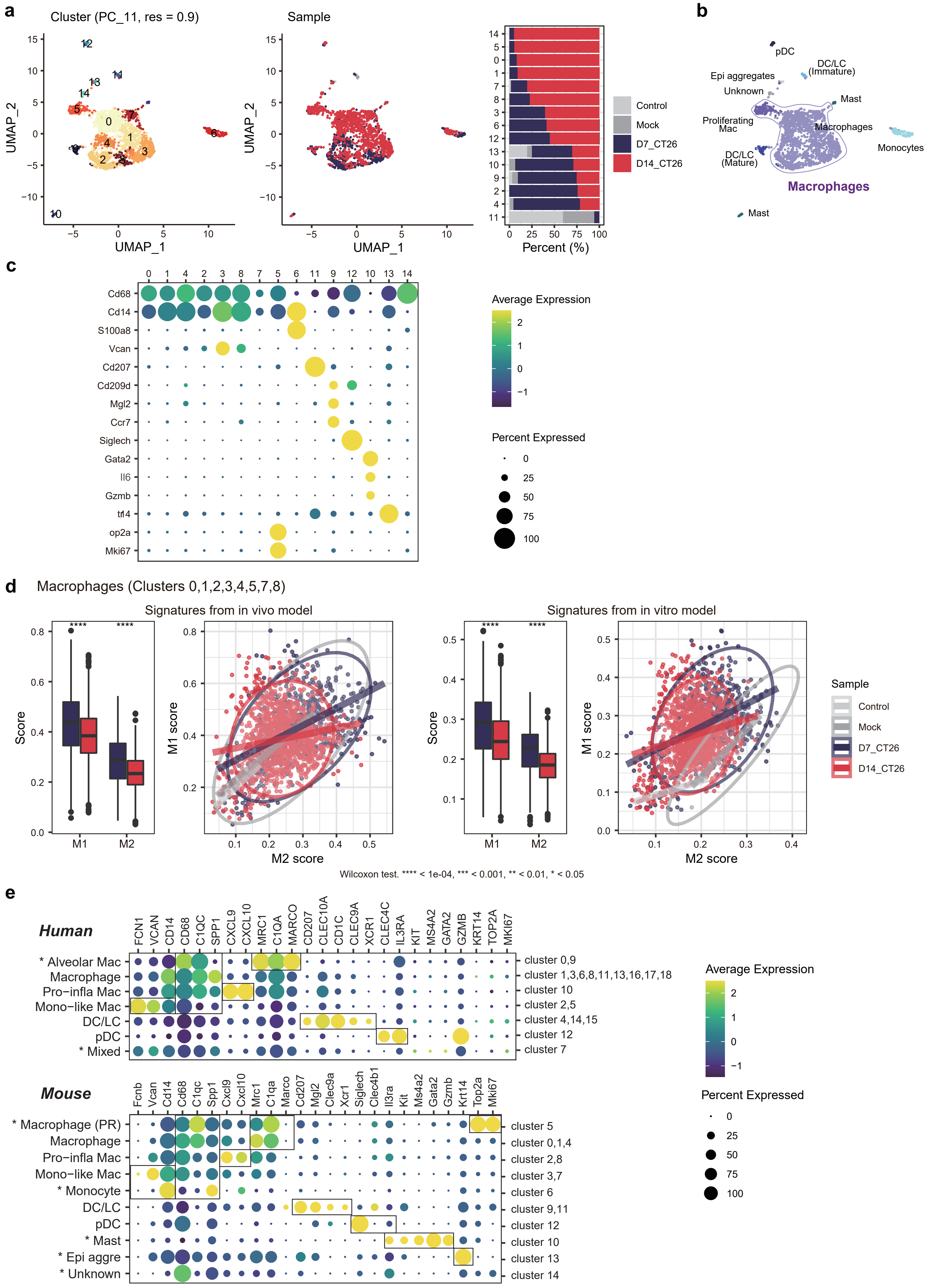

Supplementary Fig 6. Analysis of myeloid subset using scRNA-seq during tumor growth. a, UMAP plots of myeloid cells (left) and corresponding bar plot of sample proportions in each cluster (right). UMAP plots were colored by cluster or sample as indicated. **b,** UMAP plot colored with subsets of myeloid cells. **c,** Dot plot of expression of known cell subset markers in each cluster. Color depicts mean expression value and size depicts the fraction of cells with the gene expression. **d,** Comparison of M1 and M2 signatures between time points (D7 and D14). The M1/M2 signatures obtained from *in vivo* model (left) or *in vitro* model (right) were used. In boxplot, significance of differences in scores was tested with the Wilcoxon test. A linear regression model was applied to represent the macrophage characteristic axis of each sample. **e,** Dot plots of human and mouse data for known marker genes of each myeloid subset. Box indicates subset-specific marker expression and asterisk (*) indicates a subcluster detected in only one species.

Supplementary Figure 7

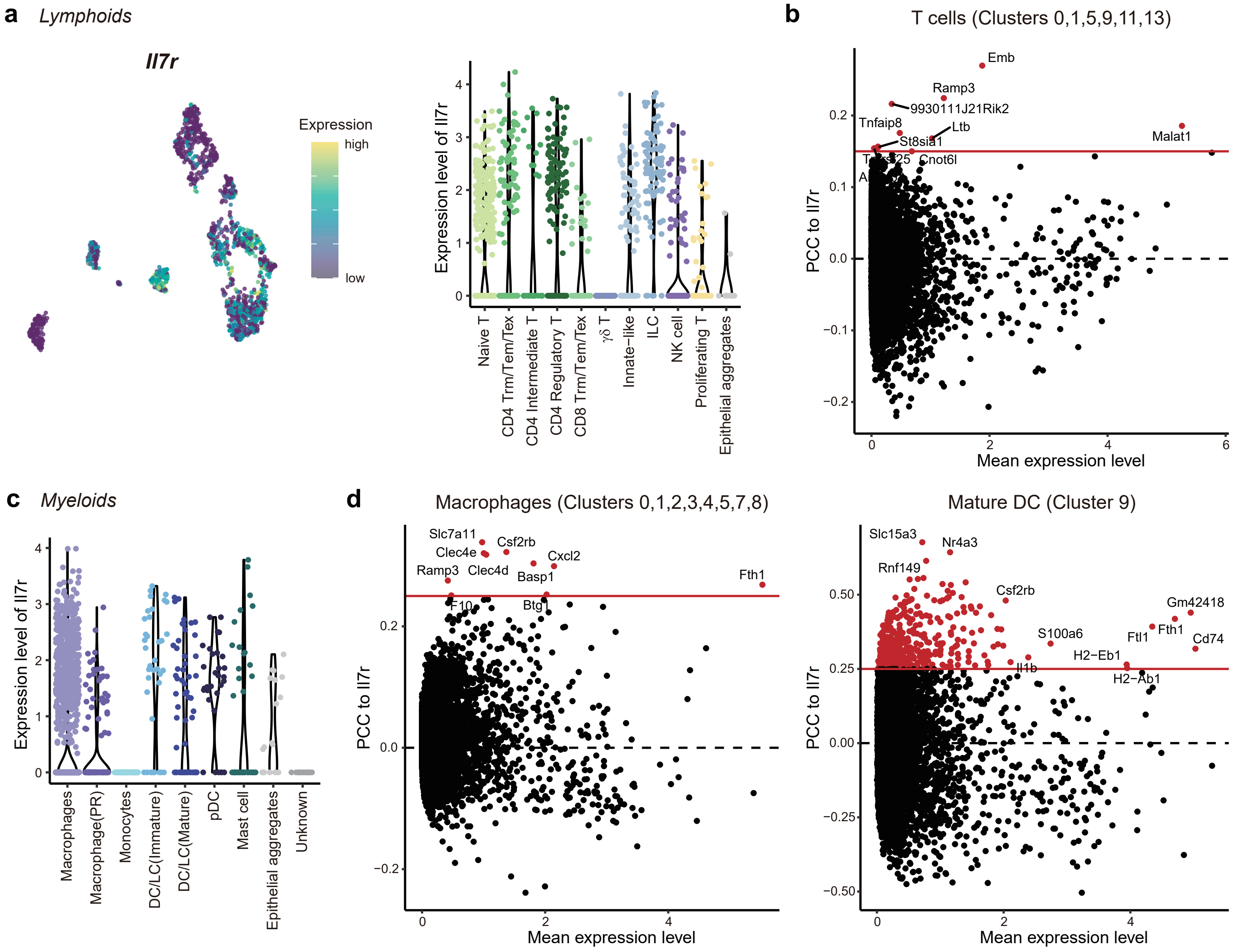

Supplementary Fig 7. Expression of Il7r gene in scRNAseq data of mouse model. a, Expression of *Il7r* in lymphoid cells. UMAP plot was colored by gene expression level (left). Violin plot shows gene expression level in each subset (right). **b,** Correlation analysis between the expression of *Il7r* and that of other genes in T cells. The x-axis and y-axis indicate the average expression of the gene and Pearson's correlation coefficient (PCC) to *Il7r* expression, respectively. **c,** Expression of *Il7r* in my-eloid cells. Violin plot shows gene expression level in each subset. **d,** Correlation analysis between the expression of *Il7r* and that of other genes in macrophages (left) and mature dendritic cells (right). The x-axis and y-axis indicate the average expression of the gene and Pearson's correlation coefficient (PCC) to *Il7r* expression, respectively.

Supplementary Figure 8

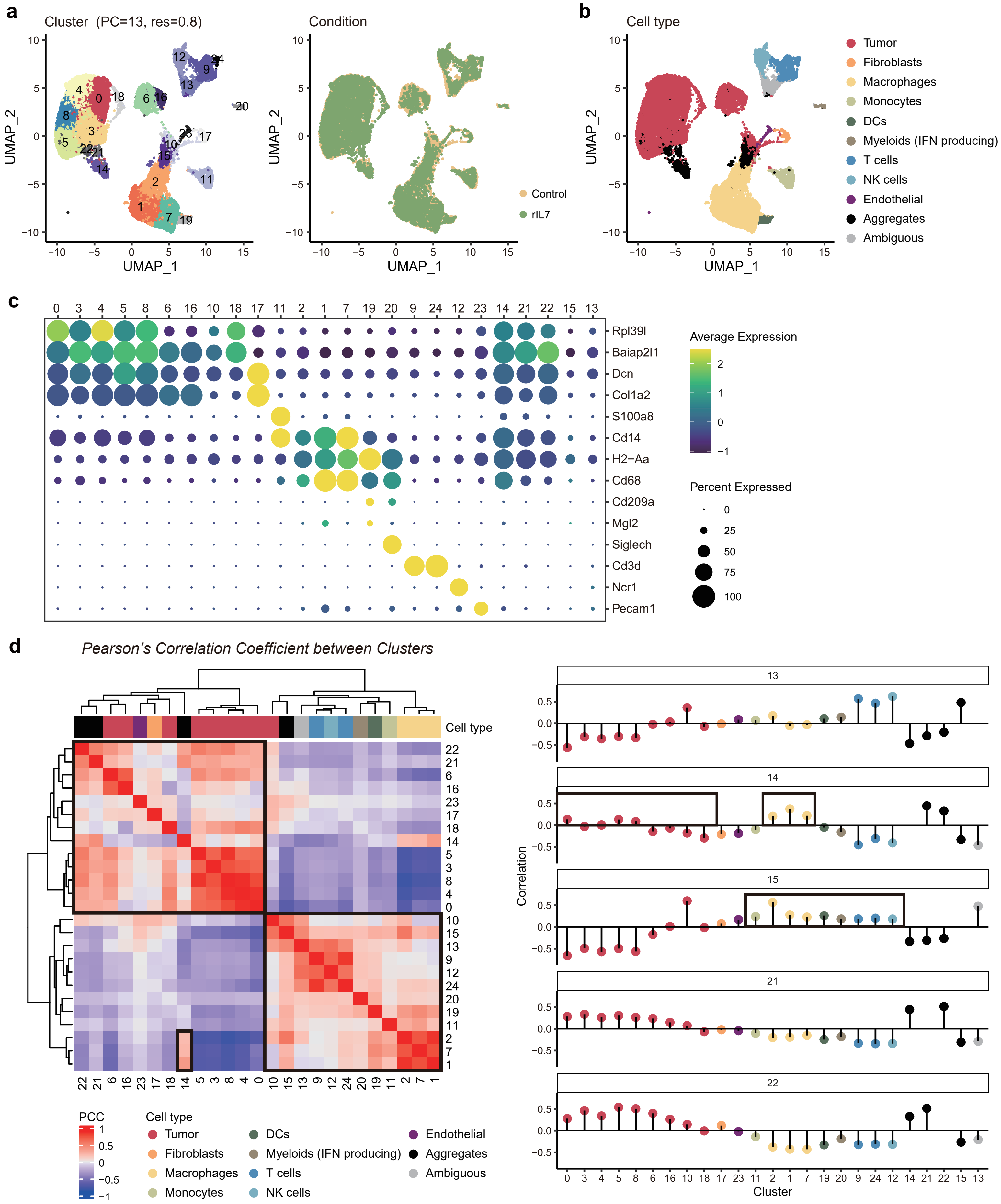

Supplementary Fig 8. scRNAseq of mouse tumor model with rIL7 treatment. a, UMAP plots of 22,756 cells, colored by cluster or treatment condition (sample) as indicated. **b,** UMAP plot color annotated by cell types. **c,** Dot plot of expression of known cell subset markers in each cluster. Color depicts mean expression value and size depicts the fraction of cells with the gene expression. **d,** Correlation plots between clusters illustrate similar cell types with aggregates (cluster 14, 15, 21, and 22) or ambiguous cluster (cluster 13).

Supplementary Figure 9

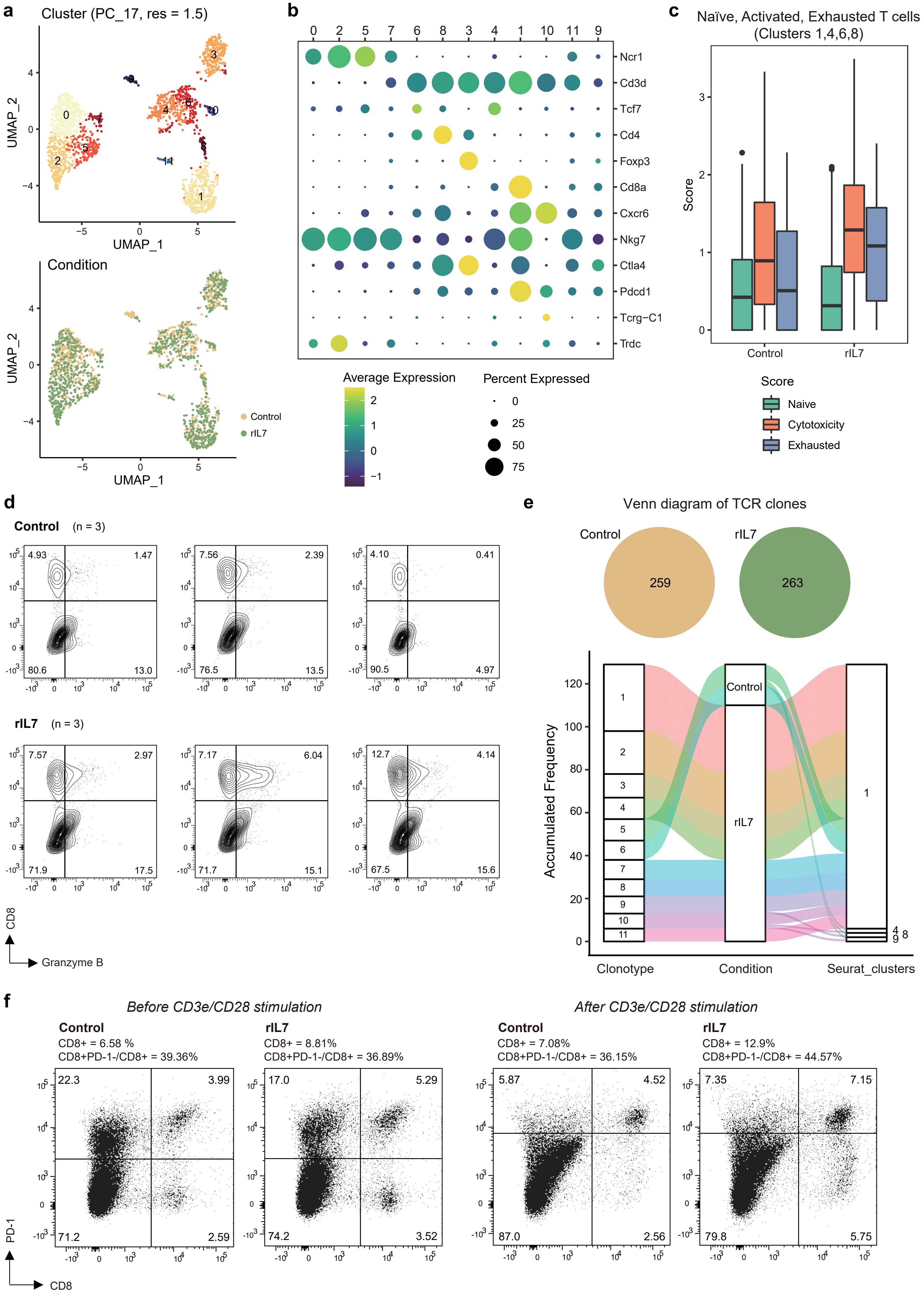

Supplementary Fig 9. Analysis of T/NK subset using scRNA-seq after rIL7 treatment. a, UMAP plots of T/NK cells, colored by cluster, treatment condition (sample), and subset as indicated. **b,** Dot plot of expression of known cell subset markers in each cluster. Color depicts mean expression value and size depicts the fraction of cells with the gene expression. **c,** Boxplot of T cell functionality scores in each condition. **d,** Flow cytometry analysis of CD8+ GranzymeB+ cells in the CD45+ CD3+ fraction of individual mouse in relation to Fig. 3g. **e,** Venn diagram (upper) and alluvial plot (lower) indicate that there were no overlapping TCR clones between control and rIL7 groups. Alluvial plot was drawn for clonotypes with more than 5 copies. **f,** Flow cytometry analysis of CD8+ T cells in rIL7 and control group. For T cell activation, CD45+ cells were incubated with CD3ε/CD28 antibodies for 21 h along with brefeldin A for the final 4 h. Numbers within quadrant indicate the percentage of cells.

Supplementary Figure 10

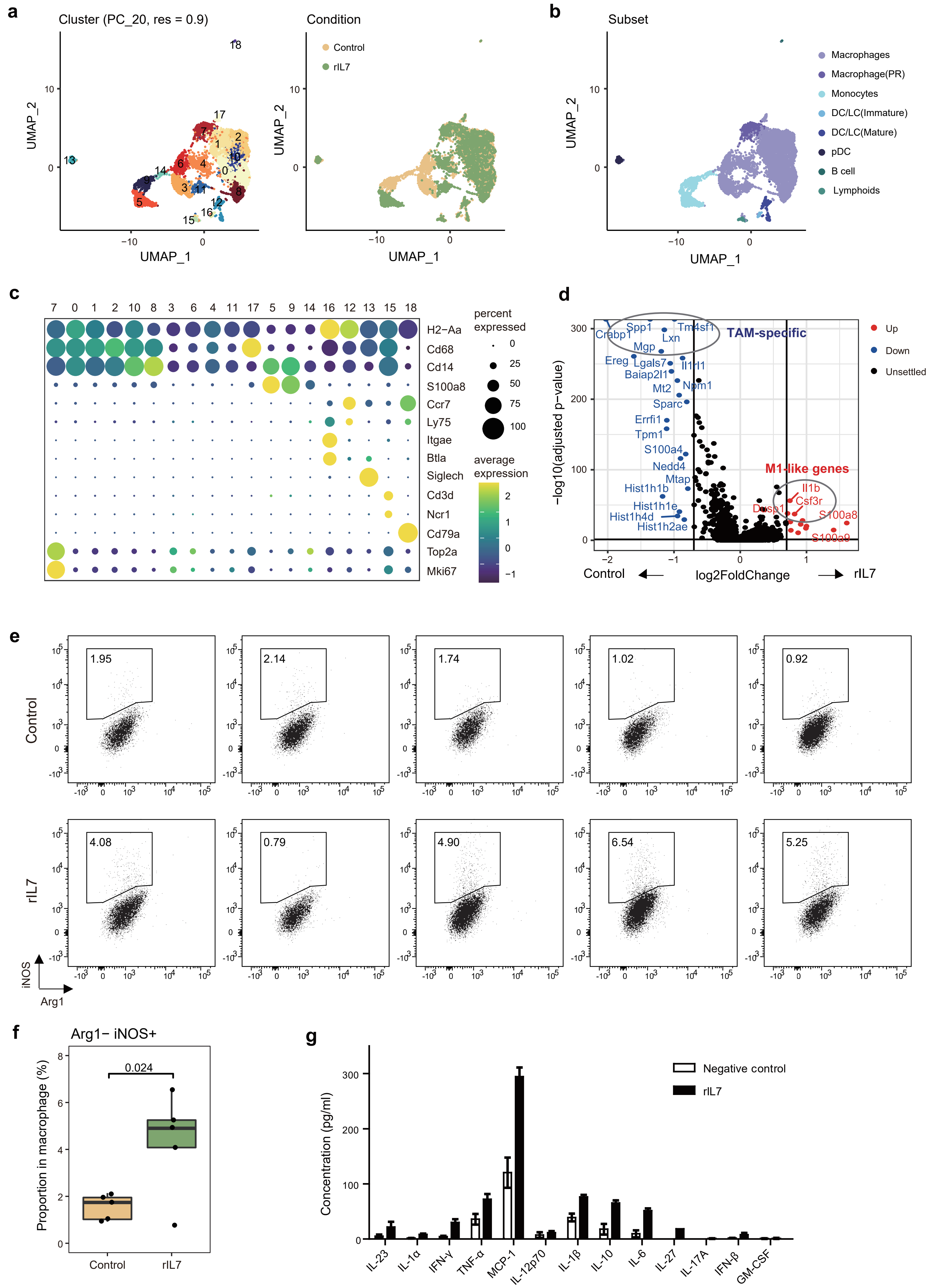

Supplementary Fig 10. Analysis of myeloid subset using scRNA-seq after rIL7 treatment. a, UMAP plots of myeloid cells, colored by cluster or condition (sample) as indicated. **b,** UMAP plot with subsets of myeloid cells. **c,** Dot plot of known cell subset markers in each cluster. Color depicts mean expression value and size depicts the fraction of cells with the gene expression. **d,** Volcano plot for differentially expressed genes between macrophages from the control and rIL7 groups. **e and f** , Flow cytometry analysis of Arg-iNOS+ cells in the CD45+ F4/80+ fraction for individual mice (e) and a summary boxplot (f). p-value from the two-sided T-test is indicated. **g,** Cytokine/chemokine levels upon in vitro rIL7 treatment of CD11b+ cells from tumor tissues (n = 3). Supernatants were analyzed by multi-analyte flow assays of IL-23, IL-a, IFN-g, TNF-a, MCP-1, IL-12p70, IL-1b, IL-10, IL-6, IL-27, IL-17A, IFN-b, and GM-CSF.

Supplementary Figure 11.

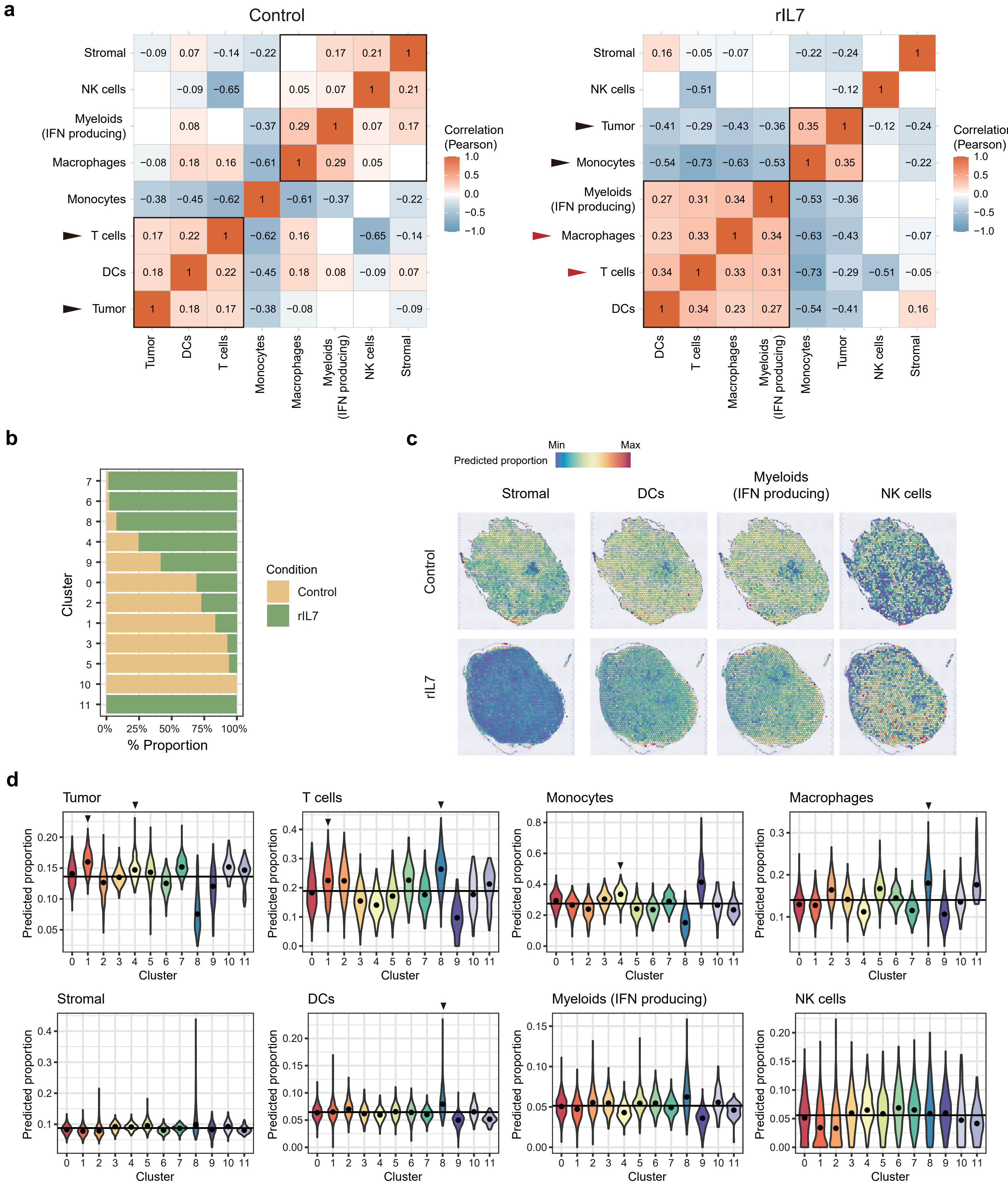

Supplementary Fig 11. Visium data of mouse tumor model with rIL7 treatment. a, Predicted cell-cell proportion correlation was analyzed by SPOTlight in each sample. Changes in T cell neighborhoods were observed (triangle annotation). **b,** Percentage bar plot of treatment condition in each cluster. **c,** Tissue images overpainted by the predicted proportion of stromal, DCs, IFN producing myeloids, and NK cells relative to Fig. 5f. **d,** Violin plots of expected cell type proportions in each cluster. Black dots and black lines represent medians within clusters and medians across clusters, respectively. Cell types co-localized in C1, C4, and C8 were annotated by triangle.
