# Supplementary material for: Single-cell RNA sequencing reveals myeloid and T cell co-stimulation mediated by IL-7 anti-cancer immunotherapy
Source: Br J Cancer. 2024 Feb 29;130(8):1388–401. doi: 10.1038/s41416-024-02617-7 (PMC11014989; doi:10.1038/s41416-024-02617-7)
Supplement: Supplementary file 3 — Supplementary Table 2 [file 41416_2024_2617_MOESM3_ESM.docx]

**Supplementary Table 2. List of antibodies used for flow cytometry, IHC, and IF.**

**Flow cytometry**

| **Target** | **Vendor** | **Catalog No** | **Clone** | **Conjugate** |
| --- | --- | --- | --- | --- |
| CD45 | BioLegend | 103108 | 30-F11 | FITC |
| CD45 | BioLegend | 103112 | 30-F11 | APC |
| CD45 | BioLegend | 103126 | 30-F11 | Pacific blue |
| CD3 | BioLegend | 100206 | 17A2 | PE |
| CD3 | BioLegend | 100204 | 17A2 | FITC |
| CD4 | BioLegend | 100412 | GK1.5 | APC |
| CD8a | BioLegend | 100738 | 53-6.7 | BV421 |
| CD8a | BioLegend | 100734 | 53-6.7 | PerCP/Cy5.5 |
| CD11b | BioLegend | 101208 | M1/70 | PE |
| CD11c | BioLegend | 117308 | N418 | PE |
| F4/80 | BioLegend | 123107 | BM8 | FITC |
| IL-7R | BioLegend | 135024 | A7R34 | Pacific blue |
| CD90.2 | BioLegend | 105305 | 30-H12 | FITC |
| Granzyme B | BioLegend | 515403 | GB11 | FITC |
| INOS | BioLegend | 696805 | W16030C | PE |
| PD-1 | BioLegend | 135205 | 29F.1A12 | PE |
| ARG1 | Novus biologicals | NBP1-32731AF647 | Polyclonal | AlexaFluor647 |

**Immunohistochemistry (IHC)**

| **Target** | **Vendor** | **Catalog No** | **Clone** | **Species reactivity** | **Host species** | **Dilution** |
| --- | --- | --- | --- | --- | --- | --- |
| CD4 | Abcam | ab183685 | EPR19514 | Mouse | Rabbit | 1:200, O/N, 4°C |
| CD8 | Cell signaling | 98941S | D4W2Z | Mouse | Rabbit | 1:200, O/N, 4°C |
| CD68 | Abcam | Ab125212 | Polyclonal | Mouse | Rabbit | 1:200, O/N, 4°C |
| Ly6C | Abcam | Ab15627 | ER-MP20 | Mouse | Mouse | 1:400, O/N, 4°C |
| CD68 | Abcam | Ab213363 | EPR20545 | Human | Rabbit | 1:1000, O/N, 4°C |
| IL7R | Abnova | MAB21101 | IL7R/2751 | Human | Mouse | 1:100, O/N, 4°C |

**Immunofluorescence (IF)**

| **Target** | **Vendor** | **Catalog No** | **Clone** | **Isotype** | **Dilution** | **Conjugate** |
| --- | --- | --- | --- | --- | --- | --- |
| CD127 | Novus Biologicals | NBP2-25249 | 73N8F2 | Rat IgG2a, k | 1:100 | Unconjugated |
| CD68 | Abcam | ab125212 | polyclonal | Rabbit IgG | 1:100 | Unconjugated |
| TNFα | R&D systems | AF-410-NA | polyclonal | Goat IgG | 1:100 | Unconjugated |
| CXCL10 | R&D systems | AF-466-NA | polyclonal | Goat IgG | 1:100 | Unconjugated |
| CD11c | BioLegend | 117311 | N418 | American Hamster IgG | 1:50 | Alexa 488 |
| **Secondary antibodies** | | | | | | |
| Rat IgG | Life technologies | A21209 | Polyclonal | Donkey IgG | 1:1000 | Alexa 594 |
| Rabbit IgG | Life technologies | A21206 | Polyclonal | Donkey IgG | 1:1000 | Alexa 488 |
| Goat IgG H&L | Abcam | ab150135 | polyclonal | Donkey IgG | 1:1000 | Alexa 647 |
